# Density‐Dependent Expression of Epitranscriptomic, Stress and Appetite Regulating Genes in Atlantic Salmon

**DOI:** 10.1111/mec.70230

**Published:** 2026-01-02

**Authors:** Morgane Frapin, Laura Quispe, Joana Troka, Jenni M. Prokkola, Ossi Laurikainen, Pekka Hyvärinen, Craig R. Primmer, Tutku Aykanat, Ehsan Pashay Ahi

**Affiliations:** ^1^ Organismal and Evolutionary Biology Research Program, Faculty of Biological and Environmental Sciences University of Helsinki Helsinki Finland; ^2^ Natural Resources Institute Finland (Luke) Helsinki Finland; ^3^ Department of Environmental and Biological Sciences, Faculty of Science, Forestry and Technology University of Eastern Finland Joensuu Finland; ^4^ Natural Resources Institute Finland (Luke) Paltamo Finland

**Keywords:** hypothalamus, life‐history, m^6^A RNA methylation, semi‐natural environment, teleost

## Abstract

Intraspecific competition due to for example, density, has substantial influence on fitness dynamics and life histories, but the underlying physiological mechanisms are often complex and the molecular basis unclear. Further, designing laboratory experiments to measure physiological responses that reflect natural conditions is challenging. Here, we reared Atlantic salmon juveniles in semi‐wild conditions in two densities to investigate the molecular mechanism of density‐related changes in the hypothalamus, a key brain region regulating stress and energy homeostasis. We measured density‐dependent changes in the expression of 12 genes involved in appetite and stress regulation and 16 genes involved in post‐transcriptional regulation of gene expression via m^6^A RNA methylation. We also quantified genotype‐environment interactions between density and two major life‐history loci, *vgll3* and *six6*. We found significant density‐related differences in the expression of genes coding for corticotropin‐releasing factors, appetite stimulators and inhibitors and m^6^A RNA methylation actors. Moreover, a paralogue of an appetite inhibitor showed a density‐dependent pattern that was the opposite of what was expected. *Six6* locus was also associated with changes in the expression of epitranscriptomic markers, including two writers and one eraser. Our results highlight that individuals' response to density in natural conditions is shaped by a complex interplay between stress, appetite and epitranscriptomic pathways in the hypothalamus. In addition, the functional divergence of paralogs indicates a potential role of genome duplication shaping such a response. We emphasise the value of integrating different physiological responses at the molecular level to better understand ecological processes affected by environmental change.

## 1 Introduction

Population density is an important factor in ecology, affecting resource availability and social interactions among conspecifics (Nislow et al. 2010; Reznick 2016). An increase in population density intensifies the intraspecific competition for resources, leading to alterations in energy homeostasis and stress levels, and subsequently impaired growth and reproductive success (Auer et al. 2012; Grossman and Simon 2020; Reznick 2016). Fishes are good models for studying density‐dependent physiological responses, as density has a significant effect on their growth and survival (Grossman and Simon 2020; Matte et al. 2020). In the wild, riverine habitats are important ecosystems that are particularly affected by density‐dependent processes, due to the limited area and resources, providing a valuable case for studying soft selection and the effects of density changes (Jonsson et al. 1998). For example, the strong territorial behaviour of Atlantic salmon to occupy favourable habitats can be beneficial in poor habitats, but may not be energetically efficient in resource rich habitats (Blanchet et al. 2006; Jonsson et al. 1998; Nislow et al. 2010). In laboratory or hatchery settings, fish are reared at much higher densities and competition and food acquisition dynamics do not reflect the natural conditions. This is likely limiting individuals from displaying natural behaviours. As such, two habitats (wild vs. laboratory conditions) are likely to result in different physiological responses to density changes (Blanchet et al. 2006; Höjesjö et al. 2004).

An individual's response to density may invoke multiple physiological and/or stress pathways, which can result in complex responses at the population level (Birk et al. 2020; Hodgson et al. 2017), with potential impacts on life histories (Archer et al. 2020). At the organismal level, such a response is regulated by the hypothalamus, which exhibits distinct molecular pathways for various environmentally induced responses (Schwartz et al. 2000; Smith and Vale 2006). The hypothalamus is a key region of the brain that connects the central nervous system to the endocrine system. It regulates feeding behaviour by balancing appetite‐stimulating genes (orexigenic genes), such as Agouti‐related protein (AgRP) and neuropeptide Y (NPY) and appetite‐suppressing genes (anorexigenic genes), such as cocaine‐ and amphetamine‐regulated transcript (CART) and pro‐opiomelanocortin (POMC). Together, these mechanisms help the body regulate energy intake and expenditure in response to nutritional status (Parker and Bloom 2012). The hypothalamus is also functioning as the primary interface for stress detection by orchestrating the glucocorticoid receptor signalling pathway and initiating the secretion of corticotropin‐releasing hormone/factor (CRH or CRF). This activates the hypothalamic–pituitary–adrenal axis, leading to the release of cortisol, a key stress hormone that prepares the body to respond to stressors (Smith and Vale 2006). Additionally, the hypothalamus plays a pivotal role in sexual maturation by releasing gonadotropin‐releasing hormone, influencing the onset of puberty and reproductive capacity (Sisk and Foster 2004). These regulatory roles of the hypothalamus are conserved across vertebrates, underscoring its fundamental importance in maintaining homeostasis and enabling responses to environmental changes and internal conditions (Denver 2009). Thus, understanding hypothalamic regulation may be critical in partitioning the relative contribution of different physiological processes, such as stress and appetite pathways, which may help to pinpoint ecological functions that underlie density‐dependent dynamics in the wild.

Emerging research highlights the role of m^6^A RNA methylation in modulating hypothalamic signals related to feeding, stress and sexual maturation in vertebrates (Ahi et al. 2024; Engel et al. 2018; Frapin et al. 2020; Yang et al. 2022). Thus, m^6^A RNA methylation may play a role in density‐dependent regulation by controlling gene expression in response to environmental changes. m^6^A RNA methylation is a widespread, reversible and evolutionarily conserved mRNA modification (Ahi and Singh 2024; Shi et al. 2022). The addition of m^6^A RNA methylation is facilitated by the m^6^A methyltransferase complex, which involves writer proteins (such as Mettl3, Mettl14 and Wtap), and removal is carried out by eraser demethylase proteins (such as Fto and Alkbh5) (Zaccara et al. 2019). Reader proteins (e.g., Ythdf1/2/3 and Ythdc1/2) recognise the m^6^A modifications, directing the methylated mRNA towards various outcomes, including degradation, stabilisation, transportation and either promotion or inhibition of translation (Liao et al. 2018; Zhao et al. 2017). Investigating whether m^6^A RNA modification affects hypothalamic signalling that controls stress and feeding in response to environmental changes, such as population density, could provide insight into growth flexibility and the proximal mechanism of phenotypic plasticity in changing environmental conditions (Jonsson et al. 1998).

The Atlantic salmon ( *Salmo salar* ) is a culturally and economically important anadromous fish species that spends its juvenile phase in freshwater. The freshwater phase is highly influenced by density effects (Thorstad et al. 2010). While molecular and/or physiological responses to density have been widely investigated in Atlantic salmon reared in artificial conditions (Liu et al. 2015; Sundh et al. 2019; Sveen et al. 2016; Wang et al. 2019), these hardly reflect natural settings, and conclusions from these studies can't necessarily be extrapolated to wild conditions. Studying the molecular/physiological pathways of density regulation is feasible in Atlantic salmon in a semi‐natural environment, and therefore more natural‐like physiological responses can be obtained. Furthermore, Atlantic salmon may also be a good model to study molecular mechanisms of life history variation and potential interplay with hypothalamic regulation of density. Genetic variants located by the *vgll3* gene in chromosome 25 and the *six6* gene in chromosome 9 have been associated with a sea age at maturity in salmon (Barson et al. 2015; Sinclair‐Waters et al. 2020). This simple genetic architecture is also associated with various behavioural, physiological and molecular pathways (e.g., Ahi et al. 2022; Aykanat et al. 2020; Bangura et al. 2022; Prokkola et al. 2022; Verta et al. 2024). A recent study reported that the hypothalamic expression of genes involved in m^6^A RNA methylation is influenced by the *six6* locus, sex and maturation status in Atlantic salmon (Ahi et al. 2024). As such, these genes may partly be associated with hypothalamic endocrine pathways that respond to environmental, dietary and physiological cues (Gerisch and Antebi 2011; Stearns 2011).

In this study, our primary aim is to investigate how changes in density affect fundamental molecular physiological processes in an environment that closely mimics natural conditions. We explore the hypothalamic expression of genes related to appetite regulation, stress hormone signalling pathways and m^6^A RNA methylation in 2‐year old Atlantic salmon parr reared in semi‐natural stream conditions, and naturally fed on mainly benthic prey items, at two different densities similar to those found naturally in the wild (Finstad et al. 2009). For stress regulation, we used a hypothesis‐driven, candidate‐gene approach focusing on corticotropin‐releasing factor (*crf*) paralogs and glucocorticoid receptors (*nr3c1* and *nr3c2*). These are core components of the hypothalamic–pituitary–interrenal axis and have been widely used as markers of stress and social interactions in teleost fishes (Bernier 2006; Chen and Fernald 2008; Conde‐Sieira et al. 2010; Lai et al. 2021). For m^6^A RNA methylation, we targeted the conserved core ‘writer’ (*mettl3*, *mettl14*, *wtap*), ‘eraser’ (*alkbh5* and *fto* paralogs) and YTH‐domain ‘reader’ families that have been annotated in Atlantic salmon and other vertebrates (Ahi et al. 2024; Ahi and Singh 2024; Zaccara et al. 2019) acknowledging that these constitute a tractable, functionally characterised subset rather than an exhaustive list of all potential m^6^A regulators. In addition, we measured total m^6^A RNA methylation percentages to assess potential, large‐scale density effect on global m^6^A RNA signatures. In addition, the presence of the genetic variation at the two loci associated with sea age at maturity, *vgll3* and *six6*, allowed us to assess their effect, as well as their interaction with density, on the gene expression of the targeted hypothalamic pathways. Given the whole‐genome duplication which occurred approximately 80 million years ago in salmonids (Dysin et al. 2022; Lien et al. 2016), we also investigate potential functional evolution of the duplicated genes by characterising the expression pattern of paralogs (Force et al. 1999; Kassahn et al. 2009).

## 2 Materials and Methods

### 2.1 Fish Rearing

All fish experiments were conducted in accordance with the Finnish Project Authorisation Board approval (ESAVI/4511/2020). In this experiment, Atlantic salmon were obtained from broodstocks reared by the Natural Resources Institute Finland (LUKE, Laukaa, Finland). The parents of the broodstocks, that is, the grandparents of the experimental fish, originated from the Kymijoki River in Southern Finland. From the broodstock, three unrelated males and females heterozygous for the alleles associated with earlier (E) and later (L) maturation at both the *vgll3* and *six6* loci were crossed in October 2019 to obtain three unrelated families, each containing offspring with all combinations of genotypes for these loci. Fertilised eggs were incubated in the dark at 7°C at the University of Helsinki until the alevin stage as described earlier (Debes et al. 2021), and then transferred late January 2020 to the Kainuu Fisheries Research Station of LUKE in Paltamo (Finland). The fish were reared in a seasonal temperature and daylight cycle and fed based on their size (Veronesi vita 0.2–0.8 mm, Raisioagro Oy) in a flow‐through system supplied with water from a nearby lake. In January 2021, fish were PIT‐tagged (12 mm) and small fin clips were taken for genotyping individuals for sex and *vgll3* and *six6* loci (Sinclair‐Waters et al. 2022). In May 2021, a subset of fish was released in six parallel outdoor channels (length: 25 m, width: 1.5 m, approx. 39 m^2^) with concrete walls and bottoms covered by a 15 cm layer of gravel‐to‐pebble‐sized particles (8–50 mm in diameter). No additional feed was provided after transfer to these outdoor channels for roughly 12 months until sampling. These semi‐natural stream channels were provided with water from the nearby lake (flow rate = 0.15–0.25 ms^−1^) and supplied the salmon with live natural prey including benthic prey items from the bottom substrate (insects, arachnids and crustaceans), and also some surface prey (such as adult midges, mosquitoes), similar to their natural habitat. Inflow of equal volume of water flow (litres/second) of previously unused water in each stream ensured similar amount and same quality of insects drifting into each stream (Rodewald et al. 2011). A large pipe (diameter 900 mm) takes water from the lake, which then was divided separately into each stream by smaller pipes (100 mm). As such, the outflow water from streams was not re‐used in any of the experimental streams. While we did not directly quantify invertebrate prey in the streams, a constant drift of insects (with the incoming water) that is distributed equally into each stream and similar gravel substrate should ensure an equal amount of available food in each stream during the whole study. All fish selected in this experiment were smolts based on silvery coloration. Each of the three families was divided between two stream channels with two density treatments, which initially had 48 (1.2 fish/m^2^) and 112 (2.8 fish/m^2^) fish per low‐density and high‐density treatment streams, respectively. However, an infection outbreak caused by *Saprolegnia* spp. right after the transfer to the outdoor tanks inflicted high mortalities to the experimental fish (as high as 46% mortality in less than a month). To bolster the density effect, high‐density tanks were supplemented with additional fish, which were not included in subsequent analysis. These additional fish originated from the same families and were reared in the same indoor tanks as the experimental fish prior to the transfer to the natural stream channels. In August 2021, due to practical reasons, all fish were transferred from the channels to six circular stream channels (Outer circumference: 26.15 m, width: 1.5 m, approx. 39.2 m^2^) with concrete walls and a bottom covered with a 15 cm layer of gravel (8–50 mm in diameter) and a gravity driven flow of 0.11 ms^−1^. The densities by the end of the experiments ranged from 0.35–0.51 fish/m^2^ and 1.62–2.05 fish/m^2^ for low‐density and high‐density stream channels, respectively (see Table S1 for details).

### 2.2 Sampling and RNA Extraction

On 25–27 April 2022, fish were caught from the streams by netting after water level in each stream was lowered and placed in aerated 40‐L buckets until sampling the same day. Handling and temporary confinement procedure was applied equally to all density treatments and genotypes, thus any additional stress due to these procedures is expected to affect all groups similarly. Fish were euthanised with an overdose of benzocaine (200 mg/L), weighed and measured. The hypothalamus of fish from both densities and all genotypes was sampled, snap‐frozen in liquid nitrogen and stored at −70°C prior to processing.

To avoid introducing variation associated with maturation status, only immature individuals were selected for this study. Maturity was assessed by checking the presence of milt in males (females are immature at that age). The hypothalamus of 81 individuals (40 males and 41 females) was selected. The individuals were either homozygous (EE or LL) or heterozygous (EL) for *six6* and *vgll3* loci (*N* = 3–10 samples per genotype, sex and density) (see Table S2 for details). Total RNA was extracted using Nucleospin RNA kit (Machery–Nagel). Homogenization and cell lysis steps were performed simultaneously by transferring the frozen hypothalamus in a 2 mL screw cap tube containing 1.4 mm ceramic beads (OMNI), 3.5 μL of DTT 1 M and 350 μL of RA1 buffer and placing the tubes in a Bead Ruptor Elite (OMNI) at 4 ms^−1^ for 3 × 20 s with a 10 s break in between. The subsequent steps followed the manufacturer's instructions and RNA was eluted in 40 μL of RNase‐free water. RNA quantity and quality were assessed using Nanodrop 2000 (Thermo Fisher Scientific) and agarose gel electrophoresis.

### 2.3 cDNA Synthesis and qPCR

Synthesis of cDNA was performed using iScript cDNA Synthesis Kit (BioRad) with 1 μg of RNA. The genes *ef1a* and *hprt1* were used as housekeeping genes (Ahi et al. 2024). The expression of six genes related to stress (*nr3c1*, *nr3c2*, *crf1a1*, *crf1a2*, *crf1b1*, *crf1b2*), six genes related to appetite regulation (*agrp1*, *cart2a*, *cart2b*, *pomca1*, *pomca2*, *npya1*) and 16 genes related to epitranscriptomic mechanisms (*mettl3*, *mettl14*, *wtap*, *alkbh5‐1*, *alkbh5‐2*, *fto‐1*, *fto‐2*, *ythdc1‐1*, *ythdc1‐2*, *ythdc2*, *ythdf1‐1*, *ythdf1‐2*, *ythdf1‐3*, *ythdf2‐1*, *ythdf2‐2*, *ythdf3*) were measured using quantitative real‐time PCR (qPCR) (genes and primer sequences, and brief functional annotations are described in Table S3). The qPCR reactions were performed in a final volume of 10 μL containing 1 μL of cDNA diluted 1:10 in nuclease‐free water, 250 nM of forward and reverse primers and 5 μL of PowerUp SYBR Green Master Mix (ThermoFisher Scientific). The qPCRs were performed on 384‐well plates using the BioRad CFX384 Real Time PCR System, with each plate measuring the expression of a single target gene across all samples that were present in triplicate. The Cqs (quantification cycle) were obtained with a manually fixed threshold located in the exponential part of the amplification. The qPCR program and primer efficiency calculation were performed as described in Ahi, Richter, et al. (2019). For each sample, the Cq values of the triplicates obtained for each gene were averaged. The ΔCq values were calculated by subtracting the geometric mean of the Cq values of the housekeeping genes, *ef1a* and *hprt1*, from the Cq values of the target genes. Then, for each gene, the ΔCq values were subtracted by the ΔCq value of the sample with the lowest expression to obtain the ΔΔCq. The relative quantifications (RQ) were calculated as 2^−ΔΔCq^, and statistical analyses used the log_2_RQ (Livak and Schmittgen 2001). The averaged Cq values derived from the triplicates for all genes and samples, along with the log_2_RQ values calculated from these Cq values, are provided in the Table S4.

### 2.4 m^6^A RNA Methylation Quantification

Quantification of m^6^A RNA methylation was performed on the 81 total RNA extracted from the hypothalamus using the EpiQuikTM m^6^A RNA Methylation Quantification Kit (Epigentek). Since one preparation kit can accommodate a maximum of 96 tests, two kits were used to include all samples in duplicate, along with negative and positive controls. Samples were randomly assigned between the two kits to account for potential batch effects in the statistical model. RNA samples were diluted to 33.33 ng/μL in RNase‐free water, and the concentration was verified with Nanodrop 2000 (Thermo Scientifc). Total m^6^A RNA methylation quantification was performed according to the manufacturer's protocol with 6 μL of the diluted total RNA (approximately 200 ng). Negative and positive controls from the kit were included in each plate. The absorbance was then read at 450 nm with the EnSpire Multimode Plate Reader (PerkinElmer). Total m^6^A RNA percentages were calculated according to the manufacturer's protocol based on the standard curve and with the amount of total RNA input adjusted based on the actual concentrations of the dilutions used.

### 2.5 Statistical Analysis

All the statistical analyses were done in R (version 4.2.2). A linear mixed effects model was fitted using the lmer function in the *lme4* package (version 1.1‐31) (Bates et al. 2015), with REML = FALSE option, using a general formula as below:

$$\text{response}\sim \text{density}\times \left(\mathrm{sex}+\text{six6}+\text{vgll3}\right)+\varepsilon_{\text{Family}}+\varepsilon_{\text{Batch}}+\varepsilon_{\text{error}}$$

In which, response is either *body mass, gene expression or m* ^*6*^ *A %*. Density was modelled as a fixed effect interacting with sex, *six6* and *vgll3* genotypes. Genotypes were coded categorically to account for non‐additivity. ε_Family_, ε_Batch_ are error terms associated with family and batch effect, and ε_error_ is the residual normal variation. ε_Batch_ was only included for the *m* ^*6*^ *A % response* to account for the kit effect. The significance of the fixed effects terms was estimated using type 1 *F*‐test using the ANOVA function in the stats package (version 4.2.2) in R. The normal distribution of the model residuals was checked using the simulateResiduals function in the *DHARMa* package (version 0.4.6, Hartig et al. 2024). *p*‐values for the gene expression analysis were adjusted for multiple comparisons per model term (i.e., density, sex, *six6* genotype, *vgll3* genotype and the interaction terms: density × sex, density × *six6*, density × *vgll3*) using the Benjamini–Hochberg method to control the false discovery rate (Benjamini and Hochberg 1995). An adjusted *p*‐value below 0.05 was considered statistically significant. For terms with significant effects, post hoc pairwise comparisons were performed using estimated marginal means (EMMs) via the *emmeans* package (version 1.8.2, Lenth et al. 2024). These pairwise comparisons allowed us to examine specific contrasts between the levels of significant variables. The *p*‐values presented in the graphs are the adjusted *p*‐values, obtained from these post hoc pairwise comparisons and corrected using the Benjamini–Hochberg method. The *p*‐values in the text correspond to the adjusted *p*‐values obtained after adjusting the ANOVA results for the false discovery rate.

To investigate potential interactions between the target genes, body mass and m^6^A percentage, we performed a correlation analysis. Pearson correlations were calculated using cor.test function (*stats* package version 4.2.2) on the residuals of the fitted body mass, gene expression and m^6^A percentage models. This analysis was carried out separately for the high‐ and low‐density groups.

## 3 Results

### 3.1 Fish Body Mass Was Influenced by Density Treatment

**FIGURE 1:**
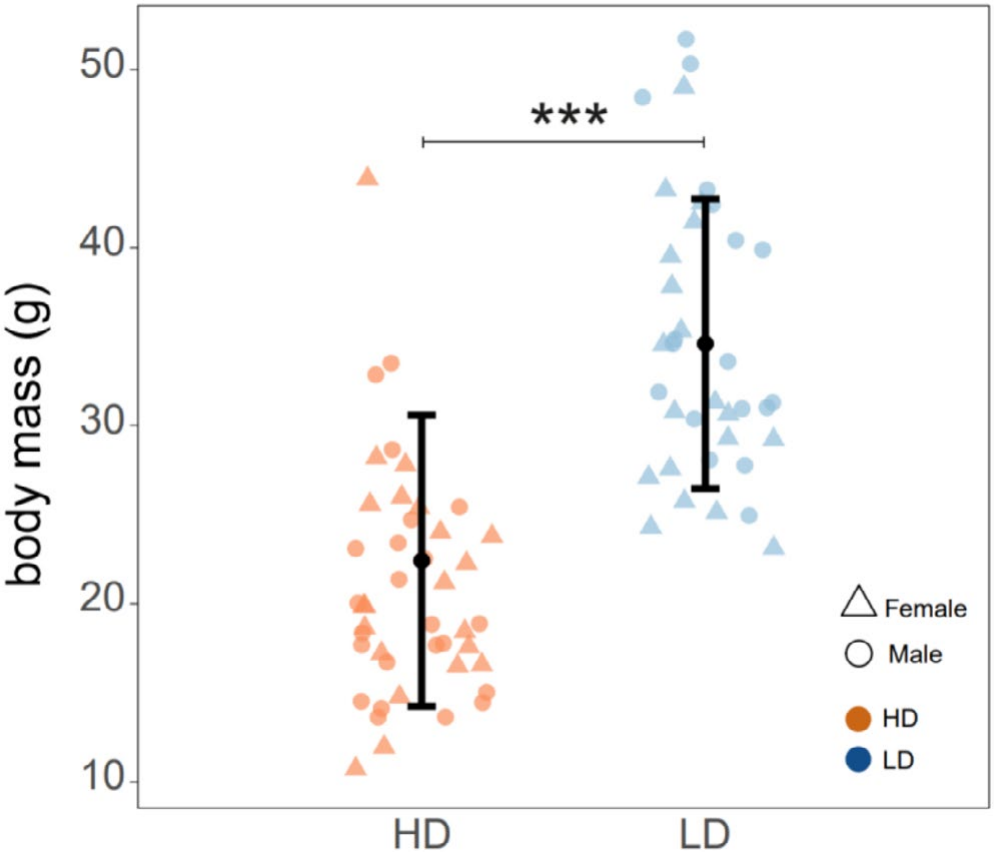
Fish body mass (in grams) measured after 1 year under density treatment. Each dot represents the body mass of an individual fish in the high‐density (HD) group (*n* = 44, orange dots) and low‐density (LD) group (*n* = 37, blue dots). The black points display the estimated marginal means for each treatment group. Error bars indicate 95% confidence intervals. ***p‐value < 0.001.

After 1 year in different densities, fish body mass was significantly lower in high‐density (*p* < 0.001; *F*_1;78.37_ = 80.425) (Figure 1). However, neither sex nor *six6* and *vgll3* genotypes had a significant effect on fish body mass.

### 3.2 Density and Genetic Factors Affect Expression Levels of Corticotropin‐Releasing Factor Genes

**FIGURE 2:**
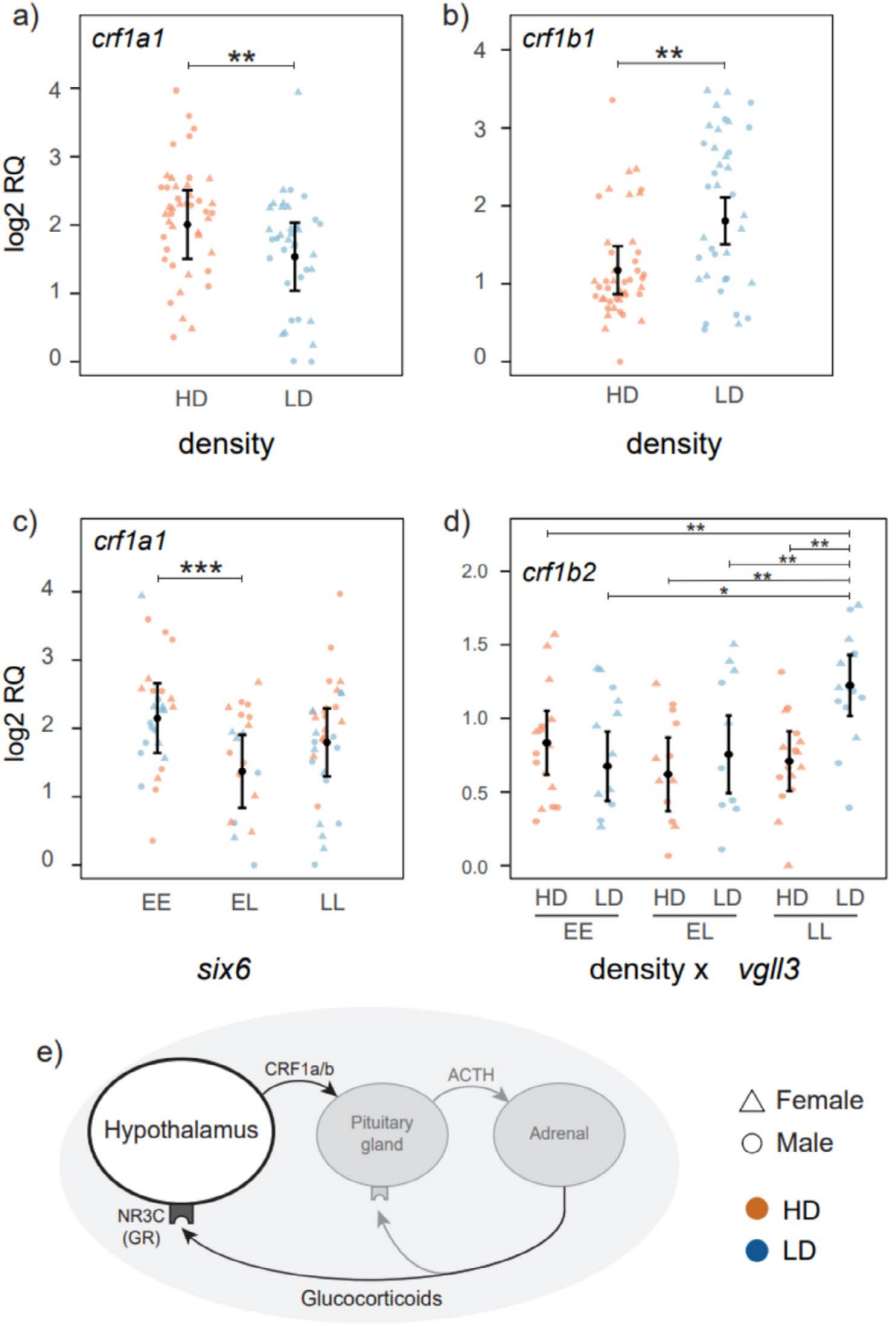
Relative mRNA expression (log_2_RQ) level of stress‐related genes affected by density (a, b), six6 (c) and the interaction between vgll3 and density (d). Error bars indicate 95% confidence intervals. *p‐value < 0.05; **p‐value < 0.01; ***p‐value < 0.001. (e) Diagram summarising the implication of the studied genes in stress regulation. Stress is sensed by the hypothalamus. This triggers a hormonal cascade initiated by the release of corticotropin‐releasing hormone/factor (CRH/CRF). Secretion of CRH stimulates the release of adrenocorticotropic hormone (ACTH) into the bloodstream, which then acts on the interrenal cells located in the fish head kidney, stimulating the release of cortisol (Gorissen and Flik 2016). Cortisol binds to glucocorticoid or mineralocorticoid receptors present in various tissues, for example, NR3C, leading to physiological and metabolic adaptations in response to the stressor and affecting fish growth (Barton and Iwama 1991; Takahashi and Sakamoto 2013)

Expression of the glucocorticoid receptor genes *nr3c1* and *nr3c2* was not significantly affected by density, sex, *six6*, or *vgll3* genotype (Table S5, Figure S1). In contrast, density and *six6* and *vgll3* genotypes influenced the expression of corticotropin‐releasing factor (*crf*) genes. The level of expression of *crf1a1* was significantly higher in the high‐density treatment (Figure 2a; *p* = 0.025; *F*_1;79.26_ = 8.598) while *crf1b1* expression was significantly lower (Figure 2b; *p* = 0.003; *F*_1;81_ = 13.801). Moreover, *crf1a1* expression was also significantly influenced by *six6* genotype (Figure 2c; *p* = 0.018, *F*_2,78_ = 6.78) with a higher level of expression in *six*6*EE (95% CI = 1.64–2.66) compared to six6*EL genotype individuals (95% CI = 0.84–1.91). An interaction effect between density and *vgll3* genotype was found for one of the corticotropin‐releasing factor paralogs, *crf1b2* (Figure 2d; *p* = 0.039, *F*_2,79_ = 7.15), with a higher level of expression of the gene in vgll3*LL in low‐density (95% CI = 1.02–1.43) compared to vgll3*EE (95% CI = 0.44–0.91) and vgll3*EL (95% CI = 0.49–1.02), and compared to vgll3*LL in high density (95% CI = 0.51–0.91).

### 3.3 Density Influences the Expression of Both Anorexigenic and Orexigenic Peptide‐Coding Genes

**FIGURE 3:**
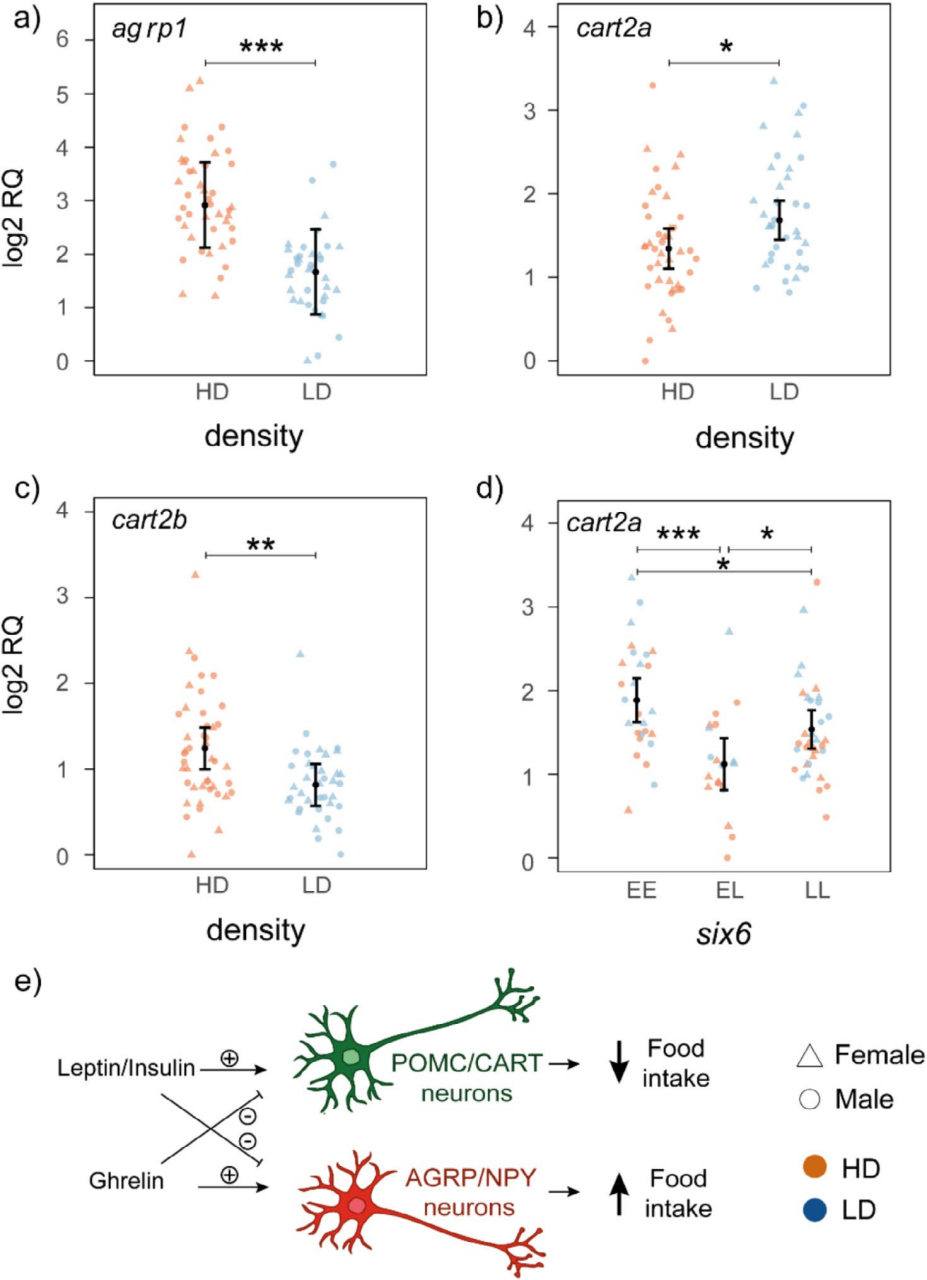
Relative mRNA expression (log_2_RQ) level of appetite‐related genes affected by density (a–c) and six6 (d). Error bars indicate 95% confidence intervals. *p‐value < 0.05; **p‐value < 0.01; ***p‐value < 0.001. (e) Diagram summarising the implication of the studied genes in appetite regulation. Orexigenic (AGRP and NPY) and anorexigenic (POMC and CART) neurons in the arcuate nucleus of the hypothalamus detect the presence of hormones (insulin, leptin and ghrelin) in the bloodstream and induce an increase (orexigenic response) or a decrease (anorexigenic response) in food intake respectively (Schneeberger et al. 2014).

The expression levels of genes encoding both appetite‐stimulating (Agrp) and appetite‐inhibiting (Cart2a/b) peptides were affected by density treatment (Table S5, Figure S1). The level of expression of *agrp1* was significantly lower at low‐density (Figure 3a; *p* < 0.001; *F*_1;79_ = 51.64). The paralogs *cart2a* (Figure 3b; *p* = 0.05; *F*_1;81_ = 6.51) and *cart2b* (Figure 3c; *p* = 0.004; *F*_1;81_ = 12.67) showed opposite patterns, with *cart2a* showing higher expression and *cart2b* lower expression at low‐density. The level of expression of *cart2a* was also influenced by *six6* genotype (Figure 3d; *p* = 0.006; *F*_2;81_ = 9.38), where heterozygote (95% CI = 0.81–1.43) exhibited significantly lower expression compared to homozygotes (95% CI = 1.62–2.15 and 1.31–1.77 for six6*EE and six6*LL, respectively).

### 3.4 Density and *six6* Genotype Affect the Expression of Genes Encoding m^6^A Regulators Without Altering Overall m^6^A Levels

**FIGURE 4:**
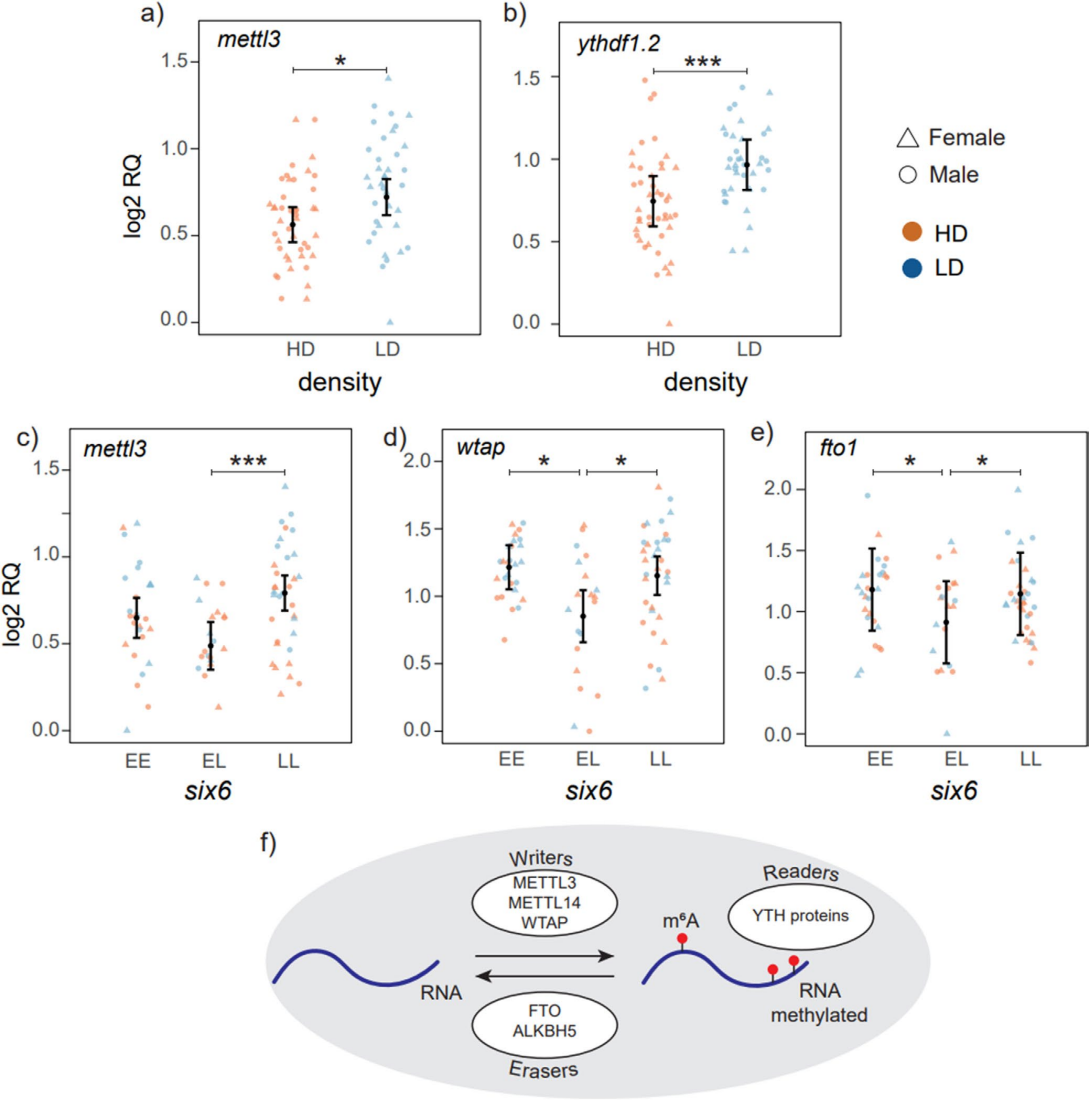
Relative mRNA expression (log_2_RQ) level of m^6^A RNA methylation‐related genes affected by density (a, b) and six6 (c–e). Error bars indicate 95% confidence intervals. *p‐value < 0.05; ***p‐value < 0.001. (f) Diagram summarising the implication of the studied genes in m^6^A RNA methylation regulation. m6A RNA methylation is added by writers (METTL3, METTL14, WTAP), forming a complex and removed by erasers (FTO and ALKBH5). The fate of RNA is determined by readers (such as the YTH family of proteins) that direct RNA to be translated, transported, or degraded (Zaccara et al. 2019).

Both density and *six6* genotype influenced the expression of genes encoding m^6^A RNA methylation writers (Table S5, Figure S1). The level of expression of *mettl3* (Figure 4a; *p* = 0.034; *F*_1;81_ = 7.55) and *ythdf1.2* (Figure 4b; *p* = 0.003; *F*_1;80_ = 14.82) was high in the low‐density group. The *six6* genotype influenced the expression levels of two writers, *mettl3* (Figure 4c; *p* = 0.006; *F*_2;81_ = 8.52) and *wtap* (Figure 4d; *p* = 0.031; *F*_2;81_ = 5.81). For *mettl3*, expression was lower in six6*EL (95% CI = 0.35–0.62) compared to six6*LL (95% CI = 0.69–0.89). *wtap* expression was reduced in six6*EL (95% CI = 0.66–1.04) compared to both six6*EE and six6*LL (95% CI = 1.05–1.38 and 1.01–1.30 for six6*EE and six6*LL, respectively). The level of expression of *fto1*, an m^6^A eraser, was also significantly affected by the *six6* genotype (Figure 4e; *p* = 0.041; *F*_2;78_ = 5.25) with a lower level of expression in six6*EL (95% CI = 0.58–1.25) compared to either of the homozygous genotypes (95% CI = 0.84–1.52 and 0.81–1.48 for six6*EE and six6*LL, respectively). The analysis of the percentage of m^6^A in total hypothalamic RNA showed no significant effects for density (*p* = 0.834; *F*_1;80_ = 0.04), sex (*p* = 0.832; *F*_1;78_ = 0.05), *six6* (*p* = 0.054; *F*_2;79_ = 3.02), and *vgll3* (*p* = 0.237; *F*_2;81_ = 1.46) genotypes.

### 3.5 Correlation Between Gene Expression Levels and m^6^A RNA Methylation Quantity

**FIGURE 5:**
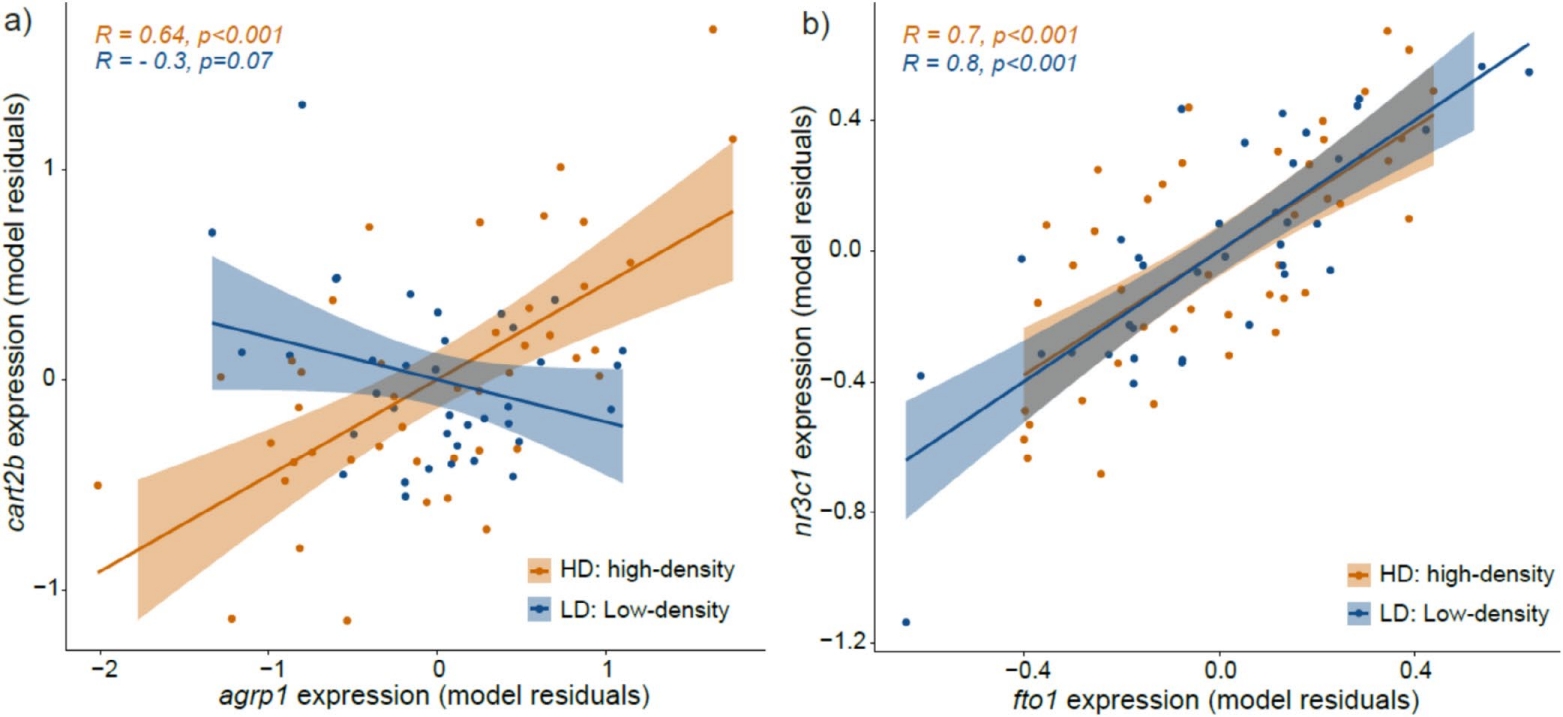
Correlation plots of residual expression levels at different densities (a) between fto1 (m^6^A‐related gene) and nr3c1 (stress‐related gene) and (b) between agrp1 and cart2b (appetite‐related genes). R: Pearson correlation coefficient.

We first examined correlations involving appetite‐related genes that responded to density, and their relationship with body mass. In the high‐density treatment, *agrp1* and *cart2b* expression levels were positively correlated (*R* = 0.64, *p* < 0.001; Figure 5a), and both genes were negatively correlated with body mass (*agrp1*–body mass: *R* = −0.56, *p* < 0.001; *cart2b*–body mass: *R* = −0.62, *p* < 0.001). No comparable pattern was observed in the low‐density group.

We next explored associations between stress‐related genes and m6A‐related markers. The expression levels of *nr3c1* and *nr3c2* were significantly correlated with several genes related to m^6^A RNA methylation in both high‐density and low‐density treatments (Figures S2 and S3). Notably, *nr3c1* expression was strongly positively associated with the level of expression of the m^6^A eraser *fto1* in both densities (*R* = 0.70 in high density and *R* = 0.80 in low density, *p* < 0.001 in both cases; Figure 5b), and *nr3c1* was negatively correlated with global m^6^A RNA methylation percentage in the low‐density group only (*R* = −0.48, *p* = 0.002).

Finally, several correlations were detected between *cart2b* and m^6^A readers and erasers, particularly under low‐density conditions. For example, *cart2b* showed negative correlations with multiple YTH‐domain readers and a positive correlation with *alkbh5.1*, and the two *alkbh5* paralogs exhibited opposite associations with m^6^A RNA methylation percentage in the low‐density treatment (*alkbh5.1*: *R* = 0.38, *p* = 0.02; *alkbh5.2*: *R* = −0.38, *p* = 0.02). The full correlation matrices for both density treatments are presented in Figures S2 and S3.

## 4 Discussion

Individuals can respond to changes in population density by adjusting appetite and stress levels (Nislow et al. 2010; Reznick 2016), both of which would be regulated via the hypothalamus. The degree of their response to density may also influence growth and, consequently, life‐history traits such as timing of maturation. However, little is known about how changes in density affect related molecular pathways in the wild. In this study, we assessed, in a semi‐natural environment, the effects of population density on the expression levels of genes related to stress and appetite, and m^6^A RNA methylation in the hypothalamus of Atlantic salmon, in relation to the genotypes of two loci associated with age at maturity (*six6* and *vgll3*). We found that density and *six6* genotype influenced the expression of several genes related to stress, appetite and m^6^A RNA methylation. A *vgll3* genotype effect was also found on a stress‐related gene (*crf1b2*) in low‐density condition. Our results also suggested widespread expression differences in paralogue gene pairs, which suggest functional divergence in these molecular pathways since the recent genome duplication event (Lien et al. 2016).

### 4.1 Density Effects on Stress‐Related Genes Suggest Functional Divergence of the *crf* Paralogs

Since population density may influence stress levels in fish by affecting food availability and social interactions, we tested the effect of density on the expression of genes encoding corticotropin‐releasing factor, CRF and glucocorticoid receptors, NR3C, which are important ubiquitous stress modulators (Figure 2e). These genes are upstream and central components of the hypothalamic–pituitary–inter‐renal stress axis in vertebrates (Bernier 2006; Chen and Fernald 2008; Conde‐Sieira et al. 2010; Lai et al. 2021). Our analysis therefore focused on these canonical regulators rather than on a genome‐wide set of stress‐related genes, and we consider this a targeted first step that can be complemented by broader transcriptomic approaches in future work. We found paralog‐specific expression differences; while *crf1a1* was upregulated in the high‐density, *crf1a2* expression was not altered, and *crf1b1* was upregulated in the low‐density group. Furthermore, in contrast to the two *crf1b* paralogs, the expression of the two *crf1a* paralogs was not significantly correlated. Previous research in aquaculture stocks has shown that the expression of *crf* increases significantly under stress conditions in the hypothalamus of rainbow trout (Conde‐Sieira et al. 2010) and in the brain of the Senegalese sole, *Solea senegalensis* (Wunderink et al. 2011). Interestingly, some studies have shown the opposite trend, with a decrease in *crf* expression under stress conditions, for example in the whole brain of zebrafish exposed to acute restraint stress (Ghisleni et al. 2012) and in a cichlid fish ( *Astatotilapia burtoni* ) after a month of social stress (Chen and Fernald 2008). This could be due to a different response depending on the stress conditions, but also to different functions of the *crf* paralogs given that the paralogue studied in teleost fish is not always clearly stated (Grone and Maruska 2015). Indeed, a recent study showed that all the *crf* paralogs (*crf1a1/2*, *crf1b1/2*) found in Atlantic salmon do not exhibit the same changes in expression patterns under stress conditions, suggesting paralog specificity (Lai et al. 2021). Lai et al. (2021) showed that hypothalamic *crf1a1* expression increased in response to hypoxia and/or hunting stress, in a pattern similar to serum cortisol levels, while *crf1a2* expression remained unchanged. The expression of the *crf1b1* and *crf1b2* paralogs was only altered during the combination of these two stress stimuli (Lai et al. 2021). This potential functional divergence of the paralogs may indicate that certain paralogs are more closely associated with the anorexigenic function previously described in teleosts (Bernier 2006), while others have a function more related to stress response.

### 4.2 *cart2b* May Have an Orexigenic Effect in High‐Density Conditions

The effect of population density was also investigated on genes involved in appetite regulation, including those encoding orexigenic peptides that stimulate appetite (*agrp* and *npy*), as well as anorexigenic peptides that inhibit appetite (*pomc* and *cart*) (Figure 3e). We found that *agrp1* showed the strongest response to density, with a highly significant, approximately twofold increase in expression at high‐density compared to low‐density. This might be due to its orexigenic role in fish and/or its responsiveness to stressors. As in many vertebrates, *agrp* has orexigenic effects in several fish species and its expression is induced in response to lack of food availability (Ahi, Brunel, et al. 2019; Cerdá‐Reverter and Peter 2003; Wei et al. 2013). Recent studies of Atlantic salmon have also shown that the hypothalamic expression of *agrp1* was increased after exposure to fasting (Kalananthan et al. 2020, 2023), suggesting a similar orexigenic role in salmonids. On the other hand, the expression of *agrp1* also significantly increased in response to stress in zebrafish (Cortés et al. 2018). Therefore, the strong induction of *agrp1* in high density conditions might be an orexigenic response due to lower food availability as well as its response to the stress related to inter‐individual competition in higher densities.

We found paralog‐specific responses of *cart* to density; *cart2b* had a significantly higher expression in high‐density, whereas *cart2a* showed an increased expression in low‐density. In zebrafish and many other teleost fishes, *cart* plays an anorexigenic role (Volkoff 2016), however, a study in zebrafish suggested that not all *cart* genes follow a similar expression pattern in response to changes in food intake (Akash et al. 2014). The increased expression of *cart2b* observed in high‐density does not support the general anorexigenic role of *cart* genes, as less food availability in high‐density is expected to reduce expression of anorexigenic genes. However, the tendency of *cart2a* towards increased expression in low‐density follows the expected anorexigenic role, thus consistent with a hypothesis that suggests a potential functional divergence for *cart2a* and *cart2b* paralogs in the hypothalamus of Atlantic salmon. Interestingly, previous studies of Atlantic salmon also revealed a similar unexpected expression pattern for the *cart2b* paralog, where its hypothalamic expression has been found significantly higher in fasted groups in response to both short‐ and long‐term fasting (Kalananthan et al. 2021, 2023). In these studies, the authors suggested a potential orexigenic role for *cart2b* in the hypothalamus of Atlantic salmon, which is uncommon across teleost fish and remains to be functionally elucidated. Moreover, in our study, *cart2b* expression was strongly correlated with the orexigenic gene, *agrp1*, but only in high‐density. Taken together, these results suggest that the neo‐functionalised *cart2b* has obtained a role that appears to be opposite to the function previously described for CART, and which may be a mechanism that helps salmon adapt to changing environmental conditions. Furthermore, both *agrp1* and *cart2b* expression levels were negatively correlated with body mass, but only under high‐density conditions. The negative correlation with *agrp1* is consistent with expectations, as reduced food availability in such conditions would likely lead to lower body mass and an increase in *agrp1* expression, a gene known to stimulate appetite. In contrast, the negative correlation with *cart2b* is unexpected for a gene that encodes an anorexigenic peptide. This finding aligns with previous observation that *cart2b* may have undergone functional divergence following genome duplication in Atlantic salmon (Kalananthan et al. 2021, 2023), resulting in a modified or divergent role from its original function.

### 4.3 Density Affects the Expression of m^6^A RNA Methylation Writer and Reader Genes

Given that m^6^A RNA methylation has been shown to be a mechanism sensitive to environmental changes (Frapin et al. 2020; Yang et al. 2022), such as variation in food availability and stress, we investigated the impact of population density on the expression of genes coding for writers, erasers and readers of m^6^A RNA methylation. Our panel targeted the conserved, core components of the m^6^A machinery (*mettl3*, *mettl14*, *wtap*, *alkbh5* and *fto* paralogs, and YTH‐domain readers), which are known to play central roles in adding, removing and interpreting m^6^A modifications (Figure 4f) (Zaccara et al. 2019), and we acknowledge that additional, less well‐characterised m^6^A regulators (e.g., *mettl5* and *IGF2BPs* (He et al. 2024; Sun et al. 2022)) were not included in this study.

We found a reduction in the hypothalamic expression of a writer, *mettl3* and a reader, *ythdf1* (*ythdf1‐2* paralog), in high‐density. This pattern is consistent with a potential involvement of these factors in the response to density‐related stress and/or food availability in the hypothalamus of Atlantic salmon, but does not in itself demonstrate that m^6^A‐mediated regulation underlies the observed changes in target gene expression. In recent years, the function of m^6^A RNA methylation factors in mediating stress signals has gained significant interest, with discoveries pointing to their versatile, tissue‐specific and context‐dependent roles (Wilkinson et al. 2021). *mettl3* is a well‐characterised and highly conserved m^6^A writer, the expression of which was reduced in high‐density compared to low‐density. Decreased *mettl3* expression under acute stress was earlier reported in the hypothalamus of mice (Engel et al. 2018). Strikingly, it seems that the stress‐mediated role of *mettl3* in the central nervous system is also highly conserved, as a similar role was proposed in the *Drosophila* brain (Perlegos et al. 2022). The second density‐affected m^6^A RNA methylation marker, *ythdf1‐2*, encodes an m^6^A reader protein that facilitates translation initiation (Wang et al. 2015). Interestingly, findings in mammals have already revealed that *ythdf1* plays a role in various stress signals such as oxidative stress, hypoxia, heat and inflammatory stresses (He et al. 2022; Lu et al. 2019; Wilkinson et al. 2021). A recent study in rainbow trout also found several *ythdf1* paralogs to be responsive to heat and inflammatory stresses in various tissues (Yu et al. 2023). Although, to our knowledge, no study has indicated its role in stress signals of social competition, a novel discovery has shown that Ythdf1 is the main mediator of microbiota‐dependent brain‐gut crosstalk in mice (Huang et al. 2023). Because m^6^A modifications can promote either stabilisation or decay of specific transcripts depending on cellular context and target mRNA (Liao et al. 2018; Zaccara et al. 2019), there was no strong a priori basis for predicting whether density would increase or decrease m^6^A levels or influence the fate of specific methylated transcripts in this context. For this reason, the density effects on *mettl3* and *ythdf1‐2* expression should be interpreted with caution. This raises a topic for future research: to understand whether *ythdf1‐2* plays a role in translating gut signals triggered by food availability to the hypothalamus in salmon kept in different densities. The answer to this question is particularly interesting because it has been already demonstrated that microbiota‐dependent brain‐gut crosstalk is essential for regulation of both appetite and body temperature (Gabanyi et al. 2022).

### 4.4 Interconnection of Stress, Appetite Regulation and m^6^A RNA Methylation Mechanism

In this study, several lines of evidence suggest that the processes/mechanisms targeted are interrelated. We observed significant correlations between nr3c paralogs and several m^6^A RNA methylation actors in both low‐ and high‐density conditions. Notably, the strongest correlation was found between the gene coding for the main glucocorticoid receptor, nr3c1, and the m6A eraser fto1, and we also detected a negative correlation between nr3c1 expression and the percentage of m^6^A RNA modifications only in the low‐density condition. Interestingly, an association between *nr3c1* and *fto1* has also been reported in recent studies, suggesting a dynamic and reciprocal regulation between FTO and NR3C1, mediated by m^6^A modifications (Roy et al. 2022; Wu et al. 2023). In our data, however, these relationships are correlational, which means that we cannot infer the direction of causality or demonstrate that m^6^A directly regulates *nr3c1* expression in response to density. Moreover, our data suggest that this regulatory mechanism could be responsive to the environment. Under high‐density condition, while a correlation between fto1 and nr3c1 was still observed, there was no significant correlation between nr3c1 and m^6^A methylation levels. Rather than indicating a confirmed ‘m^6^A‐mediated regulation’, these patterns are consistent with the possibility that external factors, such as density‐related stress, modulate both glucocorticoid signalling and components of the m^6^A machinery. This highlights the potential for a more complex, condition‐specific interaction between nr3c1 and fto that requires further investigation using gene‐specific m^6^A mapping approaches.

We did not find any clear density‐related difference in total m^6^A RNA methylation levels, suggesting that global m^6^A signatures were not altered by the density effect. However, this analysis does not rule out the possibility that m^6^A regulation at a specific locus may be linked to density but not detectable with the total m^6^A RNA assay. The observed density effect on the expression of the gene encoding the core of the writing complex, *mettl3*, and on genes previously shown to be direct targets of m^6^A RNA methylation in other vertebrates, such as CART mRNA in mice (Song et al. 2020; Wu et al. 2021) and *crh* in chicken (Yang et al. 2022), is therefore better interpreted as potential genes linking density, m^6^A machinery and hypothalamic gene expression, rather than a confirmed m^6^A‐mediated mechanism. Furthermore, we observed that *cart2b* was correlated with several m^6^A RNA readers under low‐density conditions, including paralogs of genes involved in alternative splicing (*ythdc1.2*), in fate transition (*ythdc2.1*), in mRNA clearance (*ythdf2.2*) and translation (*ythdf3*.1) (Li et al. 2017; Liao et al. 2018). This suggests that *cart2b* may be a candidate to be regulated by a variety of m^6^A‐modifying proteins, which play important roles in post‐transcriptional processes, but confirming this will require gene‐specific m^6^A mapping, such as using MeRIP‐based approaches. However, further investigation is required to understand the mechanism behind the observed difference in *cart2b* expression between the two densities.

### 4.5 Gene Expression Associations With Maturation Locus Genotypes

In Atlantic salmon, two loci near/across the genes *vgll3* and *six6* have been associated with sea age at maturity (Barson et al. 2015; Sinclair‐Waters et al. 2020). Investigating whether genotypes at these loci influence gene expression in response to environmental factors such as population density, which can affect body mass, could provide insight into how genetic and environmental factors together regulate maturation.

In this study, *six6* genotype was associated with the expression of one gene related to stress, *crf1a1*, one gene related to appetite, *cart2a*, and three genes related to m^6^A RNA methylation, *mettl3, wtap* and *fto1*. For all these genes, the six6*EL individuals had lower expression compared with six6*EE and/or six6*LL. A previous study of gene expression influenced by *six6* genotypes has only compared homozygous individuals (Ahi et al. 2024). In this study, we included heterozygotes and found notable differences in gene expression between heterozygous and homozygous individuals, indicating that six6*EL fish may have distinct hypothalamic regulatory profiles compared to *six6* homozygotes. *six6* codes for a transcription factor that is active already during development (Moustakas‐Verho et al. 2020). The genetic variation located in *six6* has been shown to exhibit strong signals of local adaptation (Pritchard et al. 2018) with demonstrated changes in the life‐history, physiological and morphological traits in Atlantic salmon (e.g., Aykanat et al. 2025; Prokkola et al. 2022; Sinclair‐Waters et al. 2020). Thus, the expression differences in heterozygotes may be associated with fitness changes. However, this hypothesis requires further investigation.

In addition, an important question for future research is to investigate whether *six6* might influence gene expression through m^6^A methylation mechanisms in a locus‐specific rather than global manner. Although no differences in overall m6A RNA methylation levels were found between the *six6* genotypes, these results do not exclude more subtle, gene‐specific differences in m6A modifications, and a more targeted analysis focusing on m6A methylation at the gene‐specific level—particularly for genes affected by the *six6* genotype—would provide more informative insights. We also found lower hypothalamic expression of *crf1b2* in the high‐density compared to low‐density condition, specifically in individuals with the vgll3*LL genotype. It has been shown in Atlantic salmon that *crf1b2* paralogue is significantly induced in the telencephalon and hypothalamus in response to chasing stress (Lai et al. 2021). Moreover, in rainbow trout, CRF administration reduces aggressive behaviours but increases anxiety‐like behaviours in a dose‐dependent manner, suggesting a dual role of CRF1 in regulating both aggression and anxiety in the brain (Backström et al. 2011). Similarly, in rodents, lower doses of CRF can induce aggression, while higher doses tend to trigger anxiety‐like responses (Hostetler and Ryabinin 2013). Notably, a recent study showed that Atlantic salmon with the vgll3*LL genotype exhibit higher aggression than those with the vgll3*EE genotype (Bangura et al. 2022). This suggests that individuals with different *vgll3* genotypes may show distinct behavioural responses under stress, such as density pressure. It is possible that *vgll3* influences aggression in salmon by differentially regulating *crf1b2* in the hypothalamus, where the balance between aggression and anxiety‐like behaviours may shift more drastically in vgll3*LL individuals under high‐density conditions due to altered *crf1b2* expression. Although no direct regulatory connection between *vgll3* (or the Hippo pathway) and *crf* genes has been identified in any species, the Hippo‐vgll3 signal may indirectly modulate *CRF* gene expression through crosstalk with stress‐related pathways, such as the glucocorticoid pathway, which acts upstream of *CRF* genes (Stepan et al. 2018).

### 4.6 Limitations and Future Directions

Although our study is among the first in exploring epitranscriptomic mechanisms in a semi‐natural setting in fish, it also has some limitations that point to fruitful directions for future work. First, we focused on a targeted panel of stress‐regulating, appetite‐controlling and m^6^A modifying genes, which does not comprise an exhaustive list of potential markers. Thus, the genes in the panel likely do not capture all transcripts that respond to density, and genome‐wide transcriptomic and epitranscriptomic approaches (e.g., MeRIP‐seq and gene‐specific m^6^A mapping) would help identify additional loci and pathways. Second, we quantified global m^6^A RNA methylation and expression of core m^6^A writers, readers and erasers, but did not assess locus‐specific m^6^A modifications on individual target genes, so any mechanistic role of m^6^A in mediating density effects on hypothalamic gene expression remains hypothetical at this stage. Third, although fish in the outdoor experimental streams were kept under semi‐natural conditions with well‐designed water distribution systems for maintaining equal natural prey abundance (see Section 2), prey availability was not directly quantified. Fourth, the sampling procedure involved short‐term confinement and handling, which may have induced acute stress responses in all fish. Since the protocol was identical across densities and genotypes, such effects are expected to add noise rather than a systematic bias, yet they may have obscured subtle treatment differences. Finally, while our sample size was sufficient to detect moderate effects of density and genotype on individual gene expression, statistical power to detect small interaction effects and subtle changes in global m^6^A levels is likely limited. Addressing these points in future studies will help to refine the mechanistic links between density, hypothalamic regulation, m6A epitranscriptomics and life‐history variation.

## 5 Conclusions

Due to the importance of Atlantic salmon in aquaculture, the effects of density on gene expression have mainly been studied under farmed conditions (Conde‐Sieira et al. 2010; Wunderink et al. 2011). Therefore, studies such as ours that use near‐natural densities can shed light on gene expression responses in more natural conditions. In this study, we found that the expression of genes related to stress, appetite and core components of the m^6^A RNA methylation machinery were affected by density under semi‐natural conditions. We did not detect density‐related differences in global m^6^A levels in the hypothalamus, and our m^6^A‐related findings should therefore be viewed as indicating that the m^6^A pathway is engaged in density responses rather than demonstrating that m^6^A causally mediates the observed changes in target gene expression. Our results suggest an interconnection between these three pathways at the level of gene expression and generate testable hypotheses about how epitranscriptomic regulation might contribute to density‐dependent physiological responses. In addition, both *six6* and *vgll3* genotypes, which are associated with sea age at maturity in Atlantic salmon, appear to influence the expression of some of the genes studied. An intriguing finding regarding the *vgll3* genotype is that it may influence anxiety‐related genes in a density‐independent manner.

Our results have broader implications for other species, especially those with comparable ecological or evolutionary pressures, by highlighting how environmental factors and genetic variation interact to influence gene expression. Furthermore, the observed interconnection between the stress and appetite regulation and m^6^A RNA methylation, which are well‐conserved mechanisms across vertebrates (Ahi and Singh 2024; Denver 2009; Rønnestad et al. 2017), provides a framework for understanding these relationships in other species while also emphasising the need for future studies that combine gene expression analyses with locus‐specific m^6^A mapping. Finally, our study highlights the importance of a deeper investigation of paralogs in salmonids and other species with recent genome duplication to better understand their specific functions, especially in adapting to environmental changes.

## Author Contributions

Experimental design (T.A., J.M.P., O.L. and P.H.). Conducting experiment (T.A., J.M.P., O.L. and C.R.P.). Genotyping (T.A. and J.M.P.). Molecular experiment design (M.F. and E.P.A.). Molecular experiment (L.Q., J.T. and M.F.). Statistical analysis (M.F. and T.A.). Funding (T.A. and C.R.P.). Writing – original draft (M.F. and E.P.A.). Writing – review and editing (M.F., T.A., E.P.A., J.M.P., C.R.P., P.H., J.T. and O.L.).

## Funding

The work was supported by C.R.P. by the Academy of Finland (grant numbers 307593, 302873, 327255 and 342851), the University of Helsinki, and the European Research Council under the European Articles Union's Horizon 2020 and Horizon Europe research and innovation programs (grant nos. 742312 and 101054307). T.A. by the Research Council of Finland (grant nos. 328860, 353388 and 325964).

## Conflicts of Interest

The authors declare no conflicts of interest.

## Supporting information


**Figure S1:** Heatmap showing the significance of model terms across genes. Each tile represents the significance of a given model term (*y*‐axis) for a specific gene (*x*‐axis). Black tiles indicate significant effects (*p* < 0.05), whereas white tiles indicate non‐significant effects. Genes on the *x*‐axis are grouped into three functional categories: stress‐related, appetite‐related and epitranscriptomic‐related.
**Figure S2:** Pearson correlation results between the model residuals of gene expression, body mass and RNA m6A methylation percentage in the high‐density condition. The values shown in the graph correspond to the Pearson correlation coefficient of the significant correlation (*p*‐value > 0.05).
**Figure S3:** Pearson correlation results between the model residuals of gene expression, body mass and RNA m6A methylation percentage in the low‐density condition. The values shown in the graph correspond to the Pearson correlation coefficient of the significant correlation (*p*‐value > 0.05).

**Table S1:** Number of fish and densities at important time points during rearing in semi‐natural environmental conditions.
**Table S2:** Number of fish used by family, sex and genotype, for the weight and the gene expression analysis.
**Table S3:** Primer information. Functional annotation is based on data obtained from GeneCards (https://www.genecards.org).
**Table S5:** Summary of Analysis of Variance (ANOVA) results.
**Table S4:** Cq values (means based on triplicate measurements) and log_2_RQ data.

## Data Availability

Data and codes are available in Figshare at https://doi.org/10.6084/m9.figshare.30719765. All the data generated in this study are included in this article as Figures S1–S3, Tables S1–S5. Benefits from this research accrue from the sharing of our data and results as described above.
