# Supplementary material for: Density‐Dependent Expression of Epitranscriptomic, Stress and Appetite Regulating Genes in Atlantic Salmon
Source: Mol Ecol. 2026 Jan 2;35(1):e70230. doi: 10.1111/mec.70230 (PMC12759207; doi:10.1111/mec.70230)
Supplement: Supplementary file 1 — Figure S1: Heatmap showing the significance of model terms across genes. Each tile represents the significance of a given model term (y‐axis) for a specific gene (x‐axis). Black tiles indicate significant effects (p < 0.05), whereas white tiles indicate non‐significant effects. Genes on the x‐axis are grouped into three functional categories: stress‐related, appetite‐related and epitranscriptomic‐related. Figure S2: Pearson correlation results between the model residuals of gene expression, body mass and RNA m6A methylation percentage in the high‐density condition. The values shown in the graph correspond to the Pearson correlation coefficient of the significant correlation (p‐value > 0.05). Figure S3: Pearson correlation results between the model residuals of gene expression, body mass and RNA m6A methylation percentage in the low‐density condition. The values shown in the graph correspond to the Pearson correlation coefficient of the significant correlation (p‐value > 0.05). [file MEC-35-e70230-s002.docx]

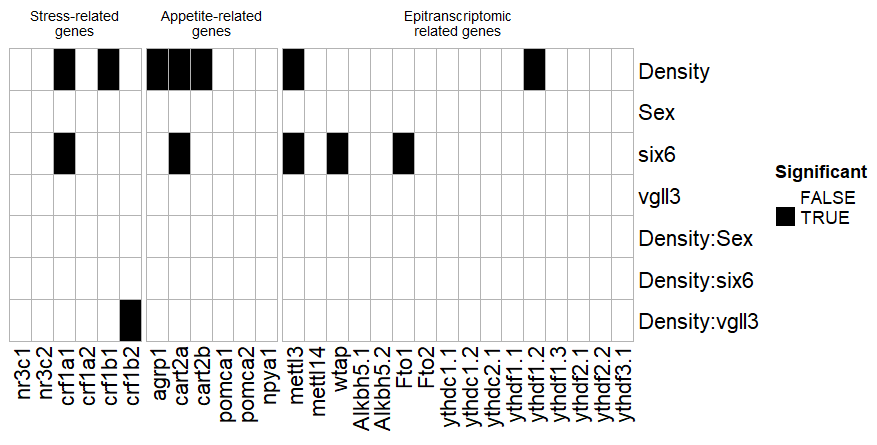


**Supplementary figure 1:** Heatmap showing the significance of model terms across genes. Each tile represents the significance of a given model term (y-axis) for a specific gene (x-axis). Black tiles indicate significant effects (p < 0.05), whereas white tiles indicate non-significant effects. Genes on the x-axis are grouped into three functional categories: stress-related, appetite-related, and epitranscriptomic-related.


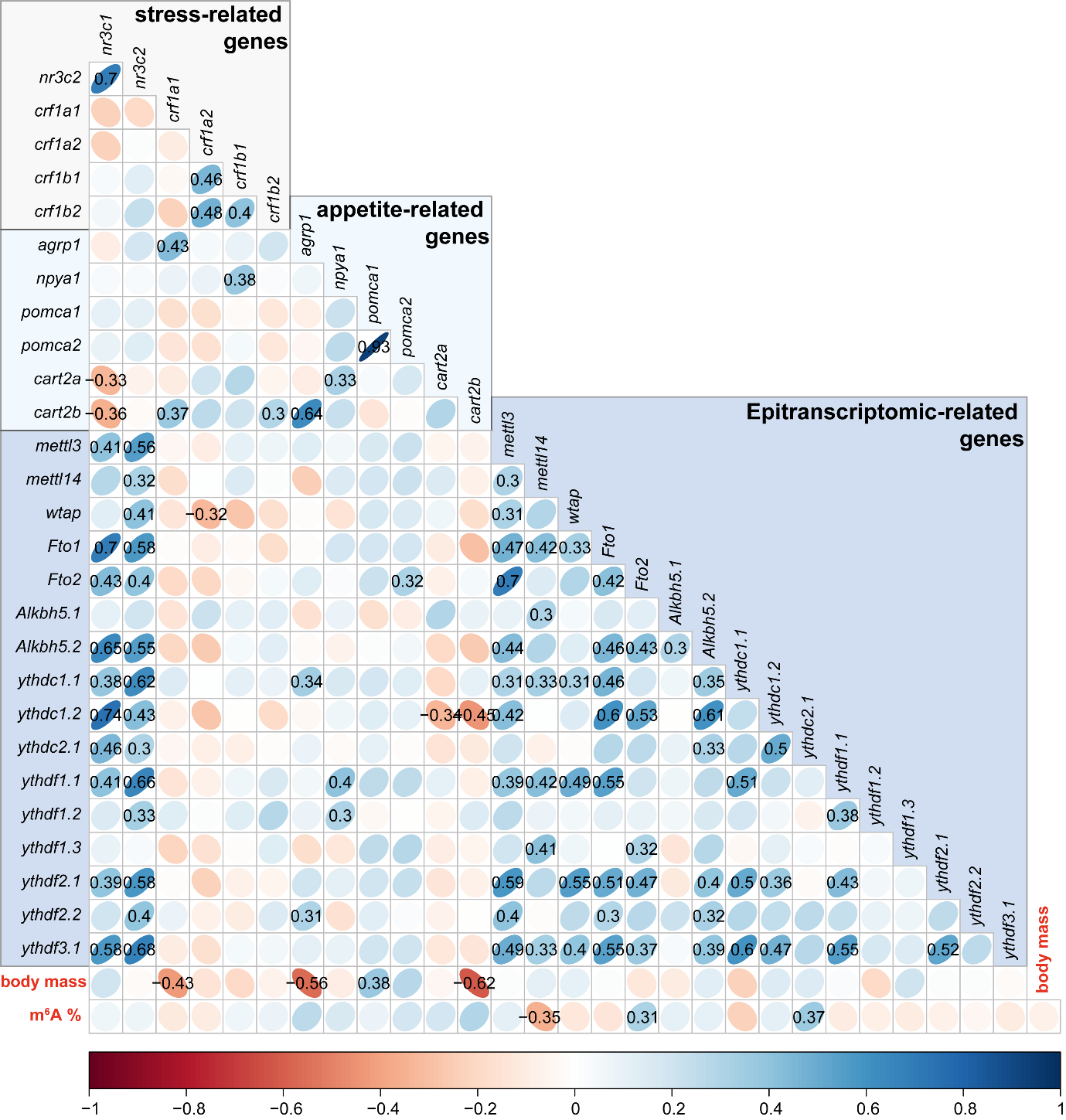


**Supplementary figure 2:** Pearson correlation results between the model residuals of gene expression, body mass and RNA m6A methylation percentage in the high-density condition. The values shown in the graph correspond to the Pearson correlation coefficient of the significant correlation (p-value > 0.05).


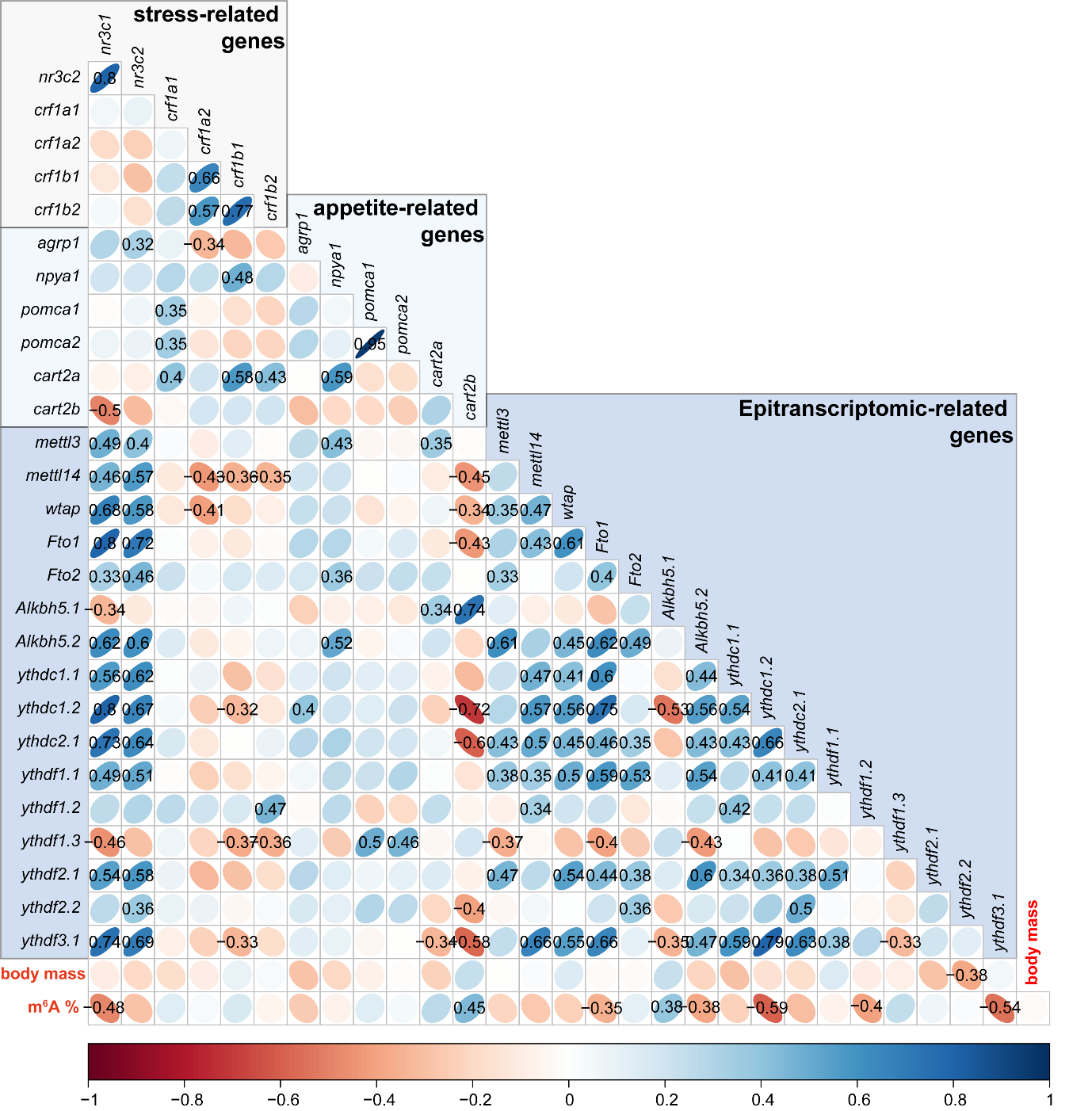
**Supplementary figure 3:** Pearson correlation results between the model residuals of gene expression, body mass and RNA m6A methylation percentage in the low-density condition. The values shown in the graph correspond to the Pearson correlation coefficient of the significant correlation (p-value > 0.05).
