# Supplementary material for: Density‐Dependent Expression of Epitranscriptomic, Stress and Appetite Regulating Genes in Atlantic Salmon
Source: Mol Ecol. 2026 Jan 2;35(1):e70230. doi: 10.1111/mec.70230 (PMC12759207; doi:10.1111/mec.70230)
Supplement: Supplementary file 2 — Table S1: Number of fish and densities at important time points during rearing in semi‐natural environmental conditions. Table S2: Number of fish used by family, sex and genotype, for the weight and the gene expression analysis. Table S3: Primer information. Functional annotation is based on data obtained from GeneCards (https://www.genecards.org). Table S5: Summary of Analysis of Variance (ANOVA) results. Table S4: Cq values (means based on triplicate measurements) and log2RQ data. [file MEC-35-e70230-s001.zip › mec70230-sup-0002-TableS1-S5.docx]

**Supplementary table 1:** Number of fish and densities at important time points during rearing in semi-natural environmental conditions.

|  |  | **Density** |  |  |  |  |  |  |
| --- | --- | --- | --- | --- | --- | --- | --- | --- |
|  |  | **Low-density** | | |  | **High-density** | | |
|  |  | **Family^1^** |  |  |  |  |  |  |
| **Date** | **Event** | **F3** | **F5** | **F8** |  | **F3** | **F5** | **F8** |
| 01 June 2021 | Fish transferred to stream channels (39 m^2^) | 48 | 48 | 48 |  | 112 | 112 | 112 |
|  | Density (m^2^) | 1.21 | 1.21 | 1.21 |  | 2.84 | 2.84 | 2.84 |
| 24 June 2021 | Number of dead fish after peak outbreak | 27 | 9 | 15 |  | 86 | 33 | 52 |
|  | Extra fish added (to enhance for density effects)^3^ |  |  |  |  | 74 | 22 | 41 |
|  | Total fish | 21 | 39 | 33 |  | 100 | 111 | 101 |
|  | Density (m^2^) | 0.54 | 1.03 | 0.85 |  | 2.56 | 2.85 | 2.59 |
| 12 August 2021 | Transfer to the round streams (39.5 m^2^) |  |  |  |  |  |  |  |
|  | Original fish | 15 | 16 | 21 |  | 18 | 51 | 47 |
|  | Extra fish |  |  |  |  | 73 | 20 | 35 |
|  | Total fish | 15 | 16 | 21 |  | 91 | 71 | 82^2^ |
|  | Density (m^2^) | 0.38 | 0.41 | 0.53 |  | 2.30 | 1.80 | 2.08 |
| 25 April 2022 | Final data count sand sampling |  |  |  |  |  |  |  |
|  | Original fish | 13 | 17 | 20 |  | 17 | 47 | 42 |
|  | Extra fish |  |  |  |  | 64 | 17 | 33 |
|  | Total fish (density / m^2^) | 14 | 17 | 20 |  | 81 | 64 | 75 |
|  | Density (m^2^) | 0.35 | 0.43 | 0.51 |  | 2.05 | 1.62 | 1.90 |

1. Each family and density combination was reared in separate stream channels.

2. 12 fish was killed during transfer to the round streams.

3. Extra stream channel was supplemented by extra fish from the same family, which were reared in the same indoor tanks prior to the transfer of experimental fish to the natural stream channels.

**Supplementary table 2:** Number of fish used by family, sex and genotype, for the weight and the gene expression analysis.

|  |  |  | **Density** | | |  | | |
| --- | --- | --- | --- | --- | --- | --- | --- | --- |
|  |  |  | **Low-density** | | | **High-density** | | |
|  |  |  | **Family** | | |  | | |
| **Locus** | **Sex** | **Genotypes** | **F3** | **F5** | **F8** | **F3** | **F5** | **F8** |
| ***vgll3*** | **Females** | **EE** | 3 | 4 | 2 | 1 | 3 | 4 |
|  |  | **EL** | 1 | 0 | 3 | 1 | 1 | 3 |
|  |  | **LL** | 1 | 3 | 2 | 2 | 3 | 3 |
|  | **Males** | **EE** | 0 | 1 | 2 | 0 | 4 | 4 |
|  |  | **EL** | 3 | 2 | 1 | 0 | 3 | 4 |
|  |  | **LL** | 3 | 2 | 4 | 2 | 5 | 1 |
| ***six6*** | **Females** | **EE** | 1 | 3 | 3 | 0 | 2 | 3 |
|  |  | **EL** | 1 | 2 | 1 | 2 | 2 | 2 |
|  |  | **LL** | 3 | 2 | 3 | 2 | 3 | 5 |
|  | **Males** | **EE** | 2 | 2 | 3 | 1 | 4 | 3 |
|  |  | **EL** | 1 | 0 | 2 | 1 | 3 | 3 |
|  |  | **LL** | 3 | 3 | 2 | 0 | 5 | 3 |

**Supplementary table 3:** Primer information. Functional annotation is based on data obtained from GeneCards (https://www.genecards.org).

|  | **Full gene name** | **Functional annotation** | **Ensembl ID** | **Forward primers (5’ - 3’)** | **Reverse primers (5’ - 3’)** | **Chr.** |
| --- | --- | --- | --- | --- | --- | --- |
| **Housekeeping genes** | | | | | | |
| *ef1a* | elongation factor 1-alpha | Deliver aminoacyl tRNAs to the ribosome | ENSSSAG00000062937 | GCCTACCCTCCCCTTGGC | GTCACCTTGCCAGTGCTGG | ssa14 |
| *hprt1* | hypoxanthine phosphoribosyltransferase 1 | Transferase enabling purine salvage | ENSSSAG00000039030 | GACTCATCCTTGACAGGACAGAGAG | CTTGAGCACGCAGAGAGCC | ssa09 |
| **Stress-related genes** | | | | | | |
| *nr3c1* | Nuclear Receptor Subfamily 3 Group C Member 1 | Glucocorticoid receptor, mediator of stress signal | ENSSSAG00000062169 | CCAGCAGCTTTGCCAGTTCA | GACAGATCTTATGGGCGGTCC | ssa05 |
| *nr3c2* | Nuclear Receptor Subfamily 3 Group C Member 2 | Mineralocorticoid receptor | ENSSSAG00000087524 | GCCAGACAGCATGTCAAGCG | GCTGCCGCATGTAACAACC | ssa04 |
| *crf1a1* | Corticotropin-Releasing Factor | Mediators of the Hypothalamic-Pituitary–Interrenal (HPI) axis to stress response | ENSSSAG00000079049 | GGAGCACTTGATCCATTCCACAATC | GATTTATTCGACAATGAGGACTGGGG | ssa03 |
| *crf1a2* | Corticotropin-Releasing Factor |  | ENSSSAG00000069223 | GGTCCATCCATCCCACGATCTA | AGGAGATGTGTTCGGCGATGA | ssa14 |
| *crf1b1* | Corticotropin-Releasing Factor |  | ENSSSAG00000052094 | CTCCACCGCTCCACAGCC | CCTCGGGGTGCATGACTTTC | ssa19 |
| *crf1b2* | Corticotropin-Releasing Factor |  | ENSSSAG00000080751 | CGCCACCGTTCCACATCAC | GGAGCCCTCTGGATACATGC | ssa29 |
| **Appetite-related genes** | | | | | | |
| *agrp1* | agouti-related protein | Orexigenic neuropeptide (stimulate appetite) | ENSSSAG00000065164 | GGAATCCTACGATGAGGATGTTGCT | GACAGGACTGCTGGTGGG | ssa11 |
| *npya1* | neuropeptide Y |  | ENSSSAG00000040508 | GGTCAAACCCGAAACCCCC | CCTCTTCCCATACCTCTGCCT | ssa14 |
| *cart2a* | Cocaine- and amphetamine-regulated transcript | Anorexigenic neuropeptide (inhibiting food intake) | ENSSSAG00000015472 | AGTTCCCACTTGCGACGTG | AACACAATGATAGGGGATTGAATCCA | ssa10 |
| *cart2b* |  |  | ENSSSAG00000047899 | CCCTACGTGCGACGTTGG | TTCAATTCACAAGCACTTGAGCAGA | ssa11 |
| *pomca1* | proopiomelanocortin |  | ENSSSAG00000004874 | TACTTTTGAAACAGCGTGACGATGC | TGCACTCCAGGATGCTGTTC | ssa09 |
| *pomca2* | proopiomelanocortin |  | ENSSSAG00000070765 | GAAGATTTGGCGACAGGCGAAG | CAGAGCTGAGGTCATGACAGC | ssa01 |
| **Epitranscriptomic-related genes** | | | | | | |
| *mettl3* | methyltransferase 3 | m⁶A RNA writers, METTL3-METTL14 heterodimer forms a N6-methyltransferase complex | ENSSSAG00000066322 | TCGTGCTCAGGTCCAGGAG | GATGATGCGTCGGAAGTGCAG | ssa18 |
| *mettl14* | methyltransferase 14 |  | ENSSSAG00020018770 | GAATAGTCGCCTACAGGAGATACGG | ACTCTCTGCTCCCAACTGTTGAG | NA |
| *wtap* | WT1 associated protein | Regulatory subunit in the m^6^A methyltransferase complex | ENSSSAG00000074636 | GGCTGAAGGAGTCTGAGGAGAAG | TGGATCTGGGTTGTACACTCTTGC | ssa15 |
| *alkbh5-1* | alkB homolog 5 | m^6^A RNA erasers, demethylation of m^6^A mRNA | ENSSSAG00000058517 | ACAGTGATCCTGAGGCGAGG | CAACCACCTCATTTATCTTACCCTCG | ssa12 |
| *alkbh5-2* | alkB homolog 5 |  | ENSSSAG00000003588 | ATCCTGTGGAGTCCCGTGAAC | CAACCACCTCATTTATCTTCCCCTCA | ssa02 |
| *fto-1* | fat mass and obesity-associated gene |  | ENSSSAG00000053871 | ACCTGAGAGATGACCTGAACAGG | GAACACTCCGCCACTCTGTG | ssa26 |
| *fto-2* | fat mass and obesity-associated gene |  | ENSSSAG00000076575 | CCTACTACCTGAGAGGTACTAGAGCA | TCTATGGGTCAGTAGGGACACAGT | ssa11 |
| *ythdc1-1* | YTH N6-methyladenosine RNA binding protein C1 | m^6^A RNA readers, regulator of alternative splicing | ENSSSAG00000072116 | CCTATGAGAGATGGCGGGGT | ATCGGAGGACTCAGCTTCTCG | ssa13 |
| *ythdc1-2* | YTH N6-methyladenosine RNA binding protein C1 |  | ENSSSAG00000048322 | CTGGTGGTGGGCAGAGGG | CTGGGACGTATCAGCTTCTCTGTAG | ssa01 |
| *ythdc2* | YTH N6-methyladenosine RNA binding protein C2 | m^6^A RNA readers, involved in RNA processing and stability | ENSSSAG00000058261 | ACAGCCACAGACAGGTCCTC | CCCTTGTCCCTGCTGCCA | ssa11 |
| *ythdf1-1* | YTH N6-methyladenosine RNA binding protein F1 | m^6^A RNA readers, mediate mRNA degradation and cellular differentiation | ENSSSAG00000048494 | GCAGGTGATGAAGATCATCGTGG | GCCAAGGATAAATGTCTTCTACTGCTG | ssa13 |
| *ythdf1-2* | YTH N6-methyladenosine RNA binding protein F1 |  | ENSSSAG00000055205 | GGTGCTCAAGATCATCGCCACT | GGACCCTCTCTGTATTGGAGCT | ssa13 |
| *ythdf1-3* | YTH N6-methyladenosine RNA binding protein F1 |  | ENSSSAG00000071507 | GTGCTGAAGATCATCGTGGGC | CTATTGTGGTTTACTTCGGTTTTGGTG | ssa15 |
| *ythdf2-1* | YTH N6-methyladenosine RNA binding protein F2 |  | ENSSSAG00000008511 | CAATGCCTATACGGCCATGTCG | GTAAGGCATGGGAGGATCTCCA | ssa01 |
| *ythdf2-2* | YTH N6-methyladenosine RNA binding protein F2 |  | ENSSSAG00000002258 | CAATGCGTATACGGCCATGTCA | TAAGGCATGGGGGGGTCG | ssa09 |
| *ythdf3* | YTH N6-methyladenosine RNA binding protein F3 |  | ENSSSAG00000056509 | TCAGGAGGTGCCTCTGGAG | TCTATTCCGCTCCCTCCGC | ssa29 |

**Supplementary table 5:** Summary of Analysis of Variance (ANOVA) results.

|  | **Genes** | **Terms** | **Sum Sq** | **Mean Sq** | **NumDF** | **DenDF** | **F value** | **Pr(>F)** | **Adjusted Pr(>F)** |
| --- | --- | --- | --- | --- | --- | --- | --- | --- | --- |
| **Stress-related genes** | ***nr3c1*** | **Density** | 0.133 | 0.133 | 1 | 79.0 | 1.095 | 0.299 | 0.492 |
|  |  | **Sex** | 0.266 | 0.266 | 1 | 77.8 | 2.182 | 0.144 | 0.402 |
|  |  | **six6** | 0.706 | 0.353 | 2 | 78.2 | 2.899 | 0.061 | 0.155 |
|  |  | **vgll3** | 0.287 | 0.144 | 2 | 78.4 | 1.180 | 0.313 | 0.398 |
|  |  | **Density:Sex** | 0.158 | 0.158 | 1 | 78.3 | 1.299 | 0.258 | 0.481 |
|  |  | **Density:six6** | 0.116 | 0.058 | 2 | 77.8 | 0.477 | 0.622 | 0.792 |
|  |  | **Density:vgll3** | 0.060 | 0.030 | 2 | 78.1 | 0.246 | 0.783 | 0.894 |
|  | ***nr3c2*** | **Density** | 0.292 | 0.292 | 1 | 79.4 | 5.244 | 0.025 | 0.069 |
|  |  | **Sex** | 0.011 | 0.011 | 1 | 77.5 | 0.195 | 0.660 | 0.845 |
|  |  | **six6** | 0.060 | 0.030 | 2 | 78.1 | 0.539 | 0.585 | 0.755 |
|  |  | **vgll3** | 0.238 | 0.119 | 2 | 78.6 | 2.136 | 0.125 | 0.269 |
|  |  | **Density:Sex** | 0.113 | 0.113 | 1 | 78.3 | 2.030 | 0.158 | 0.369 |
|  |  | **Density:six6** | 0.079 | 0.039 | 2 | 77.5 | 0.704 | 0.498 | 0.732 |
|  |  | **Density:vgll3** | 0.058 | 0.029 | 2 | 78.0 | 0.519 | 0.597 | 0.880 |
|  | ***crf1a1*** | **Density** | 3.737 | 3.737 | 1 | 79.3 | 8.598 | 0.004 | **0.025** |
|  |  | **Sex** | 0.043 | 0.043 | 1 | 77.8 | 0.098 | 0.755 | 0.845 |
|  |  | **six6** | 5.889 | 2.945 | 2 | 78.2 | 6.775 | 0.002 | **0.018** |
|  |  | **vgll3** | 1.309 | 0.655 | 2 | 78.6 | 1.506 | 0.228 | 0.336 |
|  |  | **Density:Sex** | 0.913 | 0.913 | 1 | 78.3 | 2.101 | 0.151 | 0.369 |
|  |  | **Density:six6** | 1.461 | 0.731 | 2 | 77.8 | 1.681 | 0.193 | 0.684 |
|  |  | **Density:vgll3** | 0.857 | 0.429 | 2 | 78.2 | 0.986 | 0.378 | 0.705 |
|  | ***crf1a2*** | **Density** | 0.153 | 0.153 | 1 | 81.0 | 0.228 | 0.635 | 0.720 |
|  |  | **Sex** | 3.696 | 3.696 | 1 | 81.0 | 5.490 | 0.022 | 0.107 |
|  |  | **six6** | 0.333 | 0.166 | 2 | 81.0 | 0.247 | 0.782 | 0.878 |
|  |  | **vgll3** | 2.392 | 1.196 | 2 | 81.0 | 1.777 | 0.176 | 0.308 |
|  |  | **Density:Sex** | 1.909 | 1.909 | 1 | 81.0 | 2.836 | 0.096 | 0.299 |
|  |  | **Density:six6** | 1.103 | 0.552 | 2 | 81.0 | 0.820 | 0.444 | 0.732 |
|  |  | **Density:vgll3** | 2.240 | 1.120 | 2 | 81.0 | 1.664 | 0.196 | 0.705 |
|  | ***crf1b1*** | **Density** | 6.967 | 6.967 | 1 | 81.0 | 13.801 | 0.000 | **0.003** |
|  |  | **Sex** | 3.137 | 3.137 | 1 | 81.0 | 6.215 | 0.015 | 0.107 |
|  |  | **six6** | 2.638 | 1.319 | 2 | 81.0 | 2.613 | 0.079 | 0.185 |
|  |  | **vgll3** | 4.014 | 2.007 | 2 | 81.0 | 3.976 | 0.023 | 0.090 |
|  |  | **Density:Sex** | 2.310 | 2.310 | 1 | 81.0 | 4.576 | 0.035 | 0.231 |
|  |  | **Density:six6** | 1.742 | 0.871 | 2 | 81.0 | 1.726 | 0.184 | 0.684 |
|  |  | **Density:vgll3** | 2.820 | 1.410 | 2 | 81.0 | 2.793 | 0.067 | 0.705 |
|  | ***crf1b2*** | **Density** | 0.461 | 0.461 | 1 | 80.5 | 4.314 | 0.041 | 0.104 |
|  |  | **Sex** | 1.029 | 1.029 | 1 | 78.6 | 9.622 | 0.003 | 0.075 |
|  |  | **six6** | 0.867 | 0.433 | 2 | 79.2 | 4.053 | 0.021 | 0.087 |
|  |  | **vgll3** | 1.081 | 0.540 | 2 | 80.0 | 5.051 | 0.009 | 0.074 |
|  |  | **Density:Sex** | 0.484 | 0.484 | 1 | 79.6 | 4.529 | 0.036 | 0.231 |
|  |  | **Density:six6** | 0.195 | 0.098 | 2 | 78.5 | 0.912 | 0.406 | 0.732 |
|  |  | **Density:vgll3** | 1.529 | 0.764 | 2 | 79.2 | 7.147 | 0.001 | **0.039** |
| **Appetite-related genes** | ***agrp1*** | **Density** | 26.474 | 26.474 | 1 | 78.8 | 51.640 | 0.000 | **0.000** |
|  |  | **Sex** | 0.005 | 0.005 | 1 | 78.0 | 0.011 | 0.918 | 0.918 |
|  |  | **six6** | 0.955 | 0.478 | 2 | 78.3 | 0.932 | 0.398 | 0.619 |
|  |  | **vgll3** | 0.718 | 0.359 | 2 | 78.4 | 0.701 | 0.499 | 0.583 |
|  |  | **Density:Sex** | 0.039 | 0.039 | 1 | 78.3 | 0.076 | 0.783 | 0.847 |
|  |  | **Density:six6** | 2.503 | 1.251 | 2 | 78.0 | 2.441 | 0.094 | 0.684 |
|  |  | **Density:vgll3** | 0.192 | 0.096 | 2 | 78.2 | 0.187 | 0.830 | 0.894 |
|  | ***cart2a*** | **Density** | 2.007 | 2.007 | 1 | 81.0 | 6.511 | 0.013 | **0.050** |
|  |  | **Sex** | 1.347 | 1.347 | 1 | 81.0 | 4.369 | 0.040 | 0.159 |
|  |  | **six6** | 5.783 | 2.891 | 2 | 81.0 | 9.380 | 0.000 | **0.006** |
|  |  | **vgll3** | 0.325 | 0.162 | 2 | 81.0 | 0.527 | 0.593 | 0.638 |
|  |  | **Density:Sex** | 0.675 | 0.675 | 1 | 81.0 | 2.188 | 0.143 | 0.369 |
|  |  | **Density:six6** | 0.023 | 0.011 | 2 | 81.0 | 0.037 | 0.964 | 0.964 |
|  |  | **Density:vgll3** | 0.419 | 0.209 | 2 | 81.0 | 0.679 | 0.510 | 0.793 |
|  | ***cart2b*** | **Density** | 3.122 | 3.122 | 1 | 80.5 | 12.666 | 0.001 | **0.004** |
|  |  | **Sex** | 0.219 | 0.219 | 1 | 78.7 | 0.888 | 0.349 | 0.678 |
|  |  | **six6** | 0.497 | 0.249 | 2 | 79.3 | 1.009 | 0.369 | 0.619 |
|  |  | **vgll3** | 0.823 | 0.411 | 2 | 80.1 | 1.669 | 0.195 | 0.321 |
|  |  | **Density:Sex** | 0.701 | 0.701 | 1 | 79.7 | 2.842 | 0.096 | 0.299 |
|  |  | **Density:six6** | 0.339 | 0.169 | 2 | 78.6 | 0.687 | 0.506 | 0.732 |
|  |  | **Density:vgll3** | 0.114 | 0.057 | 2 | 79.3 | 0.232 | 0.794 | 0.894 |
|  | ***pomca1*** | **Density** | 0.343 | 0.343 | 1 | 81.0 | 0.243 | 0.623 | 0.720 |
|  |  | **Sex** | 1.182 | 1.182 | 1 | 81.0 | 0.838 | 0.363 | 0.678 |
|  |  | **six6** | 3.567 | 1.783 | 2 | 81.0 | 1.264 | 0.288 | 0.538 |
|  |  | **vgll3** | 15.083 | 7.542 | 2 | 81.0 | 5.345 | 0.007 | 0.074 |
|  |  | **Density:Sex** | 4.752 | 4.752 | 1 | 81.0 | 3.368 | 0.070 | 0.299 |
|  |  | **Density:six6** | 4.964 | 2.482 | 2 | 81.0 | 1.759 | 0.179 | 0.684 |
|  |  | **Density:vgll3** | 3.240 | 1.620 | 2 | 81.0 | 1.148 | 0.322 | 0.705 |
|  | ***pomca2*** | **Density** | 1.046 | 1.046 | 1 | 81.0 | 0.651 | 0.422 | 0.591 |
|  |  | **Sex** | 0.845 | 0.845 | 1 | 81.0 | 0.525 | 0.471 | 0.759 |
|  |  | **six6** | 2.129 | 1.064 | 2 | 81.0 | 0.662 | 0.518 | 0.726 |
|  |  | **vgll3** | 17.278 | 8.639 | 2 | 81.0 | 5.375 | 0.006 | 0.074 |
|  |  | **Density:Sex** | 2.931 | 2.931 | 1 | 81.0 | 1.824 | 0.181 | 0.389 |
|  |  | **Density:six6** | 6.925 | 3.463 | 2 | 81.0 | 2.154 | 0.123 | 0.684 |
|  |  | **Density:vgll3** | 3.673 | 1.836 | 2 | 81.0 | 1.143 | 0.324 | 0.705 |
|  | ***npya1*** | **Density** | 5.097 | 5.097 | 1 | 81.0 | 5.868 | 0.018 | 0.055 |
|  |  | **Sex** | 6.881 | 6.881 | 1 | 81.0 | 7.921 | 0.006 | 0.086 |
|  |  | **six6** | 5.764 | 2.882 | 2 | 81.0 | 3.318 | 0.041 | 0.128 |
|  |  | **vgll3** | 8.378 | 4.189 | 2 | 81.0 | 4.822 | 0.011 | 0.074 |
|  |  | **Density:Sex** | 7.126 | 7.126 | 1 | 81.0 | 8.203 | 0.005 | 0.089 |
|  |  | **Density:six6** | 0.462 | 0.231 | 2 | 81.0 | 0.266 | 0.767 | 0.835 |
|  |  | **Density:vgll3** | 3.830 | 1.915 | 2 | 81.0 | 2.205 | 0.117 | 0.705 |
| **Epitranscriptomic-related genes** | ***mettl3*** | **Density** | 0.439 | 0.439 | 1 | 81.0 | 7.554 | 0.007 | **0.034** |
|  |  | **Sex** | 0.024 | 0.024 | 1 | 81.0 | 0.405 | 0.526 | 0.759 |
|  |  | **six6** | 0.989 | 0.495 | 2 | 81.0 | 8.520 | 0.000 | **0.006** |
|  |  | **vgll3** | 0.335 | 0.167 | 2 | 81.0 | 2.884 | 0.062 | 0.178 |
|  |  | **Density:Sex** | 0.012 | 0.012 | 1 | 81.0 | 0.205 | 0.652 | 0.827 |
|  |  | **Density:six6** | 0.149 | 0.074 | 2 | 81.0 | 1.281 | 0.283 | 0.732 |
|  |  | **Density:vgll3** | 0.102 | 0.051 | 2 | 81.0 | 0.877 | 0.420 | 0.735 |
|  | ***mettl14*** | **Density** | 0.351 | 0.351 | 1 | 78.9 | 2.919 | 0.091 | 0.197 |
|  |  | **Sex** | 0.683 | 0.683 | 1 | 78.0 | 5.675 | 0.020 | 0.107 |
|  |  | **six6** | 0.932 | 0.466 | 2 | 78.3 | 3.874 | 0.025 | 0.087 |
|  |  | **vgll3** | 0.322 | 0.161 | 2 | 78.5 | 1.337 | 0.269 | 0.376 |
|  |  | **Density:Sex** | 0.008 | 0.008 | 1 | 78.3 | 0.065 | 0.800 | 0.847 |
|  |  | **Density:six6** | 0.646 | 0.323 | 2 | 78.0 | 2.685 | 0.075 | 0.684 |
|  |  | **Density:vgll3** | 0.261 | 0.131 | 2 | 78.3 | 1.086 | 0.343 | 0.705 |
|  | ***wtap*** | **Density** | 0.121 | 0.121 | 1 | 81.0 | 1.018 | 0.316 | 0.492 |
|  |  | **Sex** | 0.168 | 0.168 | 1 | 81.0 | 1.417 | 0.237 | 0.604 |
|  |  | **six6** | 1.379 | 0.689 | 2 | 81.0 | 5.808 | 0.004 | **0.031** |
|  |  | **vgll3** | 0.012 | 0.006 | 2 | 81.0 | 0.049 | 0.953 | 0.988 |
|  |  | **Density:Sex** | 0.073 | 0.073 | 1 | 81.0 | 0.617 | 0.434 | 0.667 |
|  |  | **Density:six6** | 0.072 | 0.036 | 2 | 81.0 | 0.303 | 0.740 | 0.835 |
|  |  | **Density:vgll3** | 0.002 | 0.001 | 2 | 81.0 | 0.007 | 0.993 | 0.993 |
|  | ***alkbh5-1*** | **Density** | 0.584 | 0.584 | 1 | 81.0 | 1.678 | 0.199 | 0.348 |
|  |  | **Sex** | 0.009 | 0.009 | 1 | 81.0 | 0.027 | 0.870 | 0.918 |
|  |  | **six6** | 0.651 | 0.326 | 2 | 81.0 | 0.936 | 0.396 | 0.619 |
|  |  | **vgll3** | 1.098 | 0.549 | 2 | 81.0 | 1.578 | 0.213 | 0.331 |
|  |  | **Density:Sex** | 0.310 | 0.310 | 1 | 81.0 | 0.891 | 0.348 | 0.573 |
|  |  | **Density:six6** | 0.178 | 0.089 | 2 | 81.0 | 0.256 | 0.775 | 0.835 |
|  |  | **Density:vgll3** | 0.243 | 0.122 | 2 | 81.0 | 0.349 | 0.706 | 0.894 |
|  | ***alkbh5-2*** | **Density** | 0.001 | 0.001 | 1 | 80.4 | 0.019 | 0.889 | 0.889 |
|  |  | **Sex** | 0.037 | 0.037 | 1 | 78.7 | 0.837 | 0.363 | 0.678 |
|  |  | **six6** | 0.351 | 0.176 | 2 | 79.3 | 4.022 | 0.022 | 0.087 |
|  |  | **vgll3** | 0.351 | 0.176 | 2 | 79.8 | 4.021 | 0.022 | 0.090 |
|  |  | **Density:Sex** | 0.002 | 0.002 | 1 | 79.5 | 0.054 | 0.817 | 0.847 |
|  |  | **Density:six6** | 0.102 | 0.051 | 2 | 78.6 | 1.172 | 0.315 | 0.732 |
|  |  | **Density:vgll3** | 0.132 | 0.066 | 2 | 79.2 | 1.508 | 0.228 | 0.705 |
|  | ***fto-1*** | **Density** | 0.008 | 0.008 | 1 | 78.5 | 0.113 | 0.738 | 0.765 |
|  |  | **Sex** | 0.036 | 0.036 | 1 | 77.9 | 0.499 | 0.482 | 0.759 |
|  |  | **six6** | 0.759 | 0.379 | 2 | 78.1 | 5.247 | 0.007 | **0.041** |
|  |  | **vgll3** | 0.152 | 0.076 | 2 | 78.2 | 1.054 | 0.353 | 0.430 |
|  |  | **Density:Sex** | 0.097 | 0.097 | 1 | 78.1 | 1.340 | 0.251 | 0.481 |
|  |  | **Density:six6** | 0.316 | 0.158 | 2 | 77.9 | 2.184 | 0.119 | 0.684 |
|  |  | **Density:vgll3** | 0.143 | 0.072 | 2 | 78.1 | 0.991 | 0.376 | 0.705 |
|  | ***fto-2*** | **Density** | 0.030 | 0.030 | 1 | 81.0 | 0.217 | 0.643 | 0.720 |
|  |  | **Sex** | 0.469 | 0.469 | 1 | 81.0 | 3.404 | 0.069 | 0.214 |
|  |  | **six6** | 0.062 | 0.031 | 2 | 81.0 | 0.226 | 0.798 | 0.878 |
|  |  | **vgll3** | 0.564 | 0.282 | 2 | 81.0 | 2.046 | 0.136 | 0.272 |
|  |  | **Density:Sex** | 0.019 | 0.019 | 1 | 81.0 | 0.138 | 0.711 | 0.829 |
|  |  | **Density:six6** | 0.205 | 0.102 | 2 | 81.0 | 0.744 | 0.479 | 0.732 |
|  |  | **Density:vgll3** | 0.414 | 0.207 | 2 | 81.0 | 1.502 | 0.229 | 0.705 |
|  | ***ythdc1-1*** | **Density** | 0.012 | 0.012 | 1 | 80.2 | 0.121 | 0.729 | 0.765 |
|  |  | **Sex** | 0.010 | 0.010 | 1 | 77.3 | 0.098 | 0.755 | 0.845 |
|  |  | **six6** | 0.463 | 0.231 | 2 | 78.2 | 2.276 | 0.109 | 0.236 |
|  |  | **vgll3** | 0.581 | 0.290 | 2 | 79.3 | 2.857 | 0.063 | 0.178 |
|  |  | **Density:Sex** | 0.036 | 0.036 | 1 | 78.8 | 0.358 | 0.551 | 0.772 |
|  |  | **Density:six6** | 0.070 | 0.035 | 2 | 77.1 | 0.346 | 0.709 | 0.835 |
|  |  | **Density:vgll3** | 0.057 | 0.028 | 2 | 78.1 | 0.280 | 0.757 | 0.894 |
|  | ***ythdc1-2*** | **Density** | 0.031 | 0.031 | 1 | 80.6 | 0.225 | 0.637 | 0.720 |
|  |  | **Sex** | 0.168 | 0.168 | 1 | 78.4 | 1.227 | 0.271 | 0.633 |
|  |  | **six6** | 0.217 | 0.109 | 2 | 79.1 | 0.795 | 0.455 | 0.671 |
|  |  | **vgll3** | 0.724 | 0.362 | 2 | 80.3 | 2.649 | 0.077 | 0.196 |
|  |  | **Density:Sex** | 0.427 | 0.427 | 1 | 79.8 | 3.123 | 0.081 | 0.299 |
|  |  | **Density:six6** | 0.219 | 0.110 | 2 | 78.0 | 0.802 | 0.452 | 0.732 |
|  |  | **Density:vgll3** | 0.189 | 0.095 | 2 | 79.1 | 0.692 | 0.503 | 0.793 |
|  | ***ythdc2-1*** | **Density** | 0.249 | 0.249 | 1 | 79.2 | 1.696 | 0.197 | 0.348 |
|  |  | **Sex** | 0.064 | 0.064 | 1 | 77.6 | 0.438 | 0.510 | 0.759 |
|  |  | **six6** | 0.871 | 0.436 | 2 | 78.1 | 2.965 | 0.057 | 0.155 |
|  |  | **vgll3** | 0.193 | 0.096 | 2 | 78.4 | 0.656 | 0.522 | 0.584 |
|  |  | **Density:Sex** | 0.633 | 0.633 | 1 | 78.2 | 4.309 | 0.041 | 0.231 |
|  |  | **Density:six6** | 0.453 | 0.227 | 2 | 77.6 | 1.542 | 0.220 | 0.686 |
|  |  | **Density:vgll3** | 0.043 | 0.021 | 2 | 78.0 | 0.146 | 0.865 | 0.897 |
|  | ***ythdf1-1*** | **Density** | 0.257 | 0.257 | 1 | 78.6 | 5.950 | 0.017 | 0.055 |
|  |  | **Sex** | 0.007 | 0.007 | 1 | 77.8 | 0.154 | 0.696 | 0.845 |
|  |  | **six6** | 0.018 | 0.009 | 2 | 78.1 | 0.204 | 0.816 | 0.878 |
|  |  | **vgll3** | 0.388 | 0.194 | 2 | 78.2 | 4.505 | 0.014 | 0.079 |
|  |  | **Density:Sex** | 0.002 | 0.002 | 1 | 78.1 | 0.037 | 0.848 | 0.848 |
|  |  | **Density:six6** | 0.144 | 0.072 | 2 | 77.8 | 1.668 | 0.195 | 0.684 |
|  |  | **Density:vgll3** | 0.101 | 0.050 | 2 | 78.0 | 1.170 | 0.316 | 0.705 |
|  | ***ythdf1-2*** | **Density** | 0.828 | 0.828 | 1 | 79.5 | 14.820 | 0.000 | **0.003** |
|  |  | **Sex** | 0.301 | 0.301 | 1 | 77.5 | 5.387 | 0.023 | 0.107 |
|  |  | **six6** | 0.007 | 0.003 | 2 | 78.1 | 0.061 | 0.941 | 0.941 |
|  |  | **vgll3** | 0.263 | 0.132 | 2 | 78.6 | 2.358 | 0.101 | 0.236 |
|  |  | **Density:Sex** | 0.032 | 0.032 | 1 | 78.3 | 0.570 | 0.452 | 0.667 |
|  |  | **Density:six6** | 0.057 | 0.028 | 2 | 77.4 | 0.507 | 0.605 | 0.792 |
|  |  | **Density:vgll3** | 0.227 | 0.113 | 2 | 78.0 | 2.032 | 0.138 | 0.705 |
|  | ***ythdf1-3*** | **Density** | 0.094 | 0.094 | 1 | 81.0 | 0.694 | 0.407 | 0.591 |
|  |  | **Sex** | 0.051 | 0.051 | 1 | 81.0 | 0.375 | 0.542 | 0.759 |
|  |  | **six6** | 0.341 | 0.171 | 2 | 81.0 | 1.266 | 0.287 | 0.538 |
|  |  | **vgll3** | 0.001 | 0.001 | 2 | 81.0 | 0.005 | 0.995 | 0.995 |
|  |  | **Density:Sex** | 0.032 | 0.032 | 1 | 81.0 | 0.235 | 0.629 | 0.827 |
|  |  | **Density:six6** | 0.257 | 0.128 | 2 | 81.0 | 0.953 | 0.390 | 0.732 |
|  |  | **Density:vgll3** | 0.066 | 0.033 | 2 | 81.0 | 0.243 | 0.785 | 0.894 |
|  | ***ythdf2-1*** | **Density** | 0.156 | 0.156 | 1 | 81.0 | 1.852 | 0.177 | 0.348 |
|  |  | **Sex** | 0.002 | 0.002 | 1 | 81.0 | 0.020 | 0.888 | 0.918 |
|  |  | **six6** | 0.081 | 0.040 | 2 | 81.0 | 0.481 | 0.620 | 0.755 |
|  |  | **vgll3** | 0.306 | 0.153 | 2 | 81.0 | 1.815 | 0.169 | 0.308 |
|  |  | **Density:Sex** | 0.087 | 0.087 | 1 | 81.0 | 1.032 | 0.313 | 0.547 |
|  |  | **Density:six6** | 0.110 | 0.055 | 2 | 81.0 | 0.653 | 0.523 | 0.732 |
|  |  | **Density:vgll3** | 0.246 | 0.123 | 2 | 81.0 | 1.461 | 0.238 | 0.705 |
|  | ***ythdf2-2*** | **Density** | 0.064 | 0.064 | 1 | 81.0 | 0.255 | 0.615 | 0.720 |
|  |  | **Sex** | 0.042 | 0.042 | 1 | 81.0 | 0.168 | 0.683 | 0.845 |
|  |  | **six6** | 0.255 | 0.128 | 2 | 81.0 | 0.510 | 0.603 | 0.755 |
|  |  | **vgll3** | 0.612 | 0.306 | 2 | 81.0 | 1.222 | 0.300 | 0.398 |
|  |  | **Density:Sex** | 0.043 | 0.043 | 1 | 81.0 | 0.172 | 0.679 | 0.827 |
|  |  | **Density:six6** | 0.044 | 0.022 | 2 | 81.0 | 0.088 | 0.916 | 0.950 |
|  |  | **Density:vgll3** | 0.102 | 0.051 | 2 | 81.0 | 0.204 | 0.816 | 0.894 |
|  | ***ythdf3-1*** | **Density** | 0.311 | 0.311 | 1 | 81.0 | 3.926 | 0.051 | 0.119 |
|  |  | **Sex** | 0.274 | 0.274 | 1 | 81.0 | 3.456 | 0.067 | 0.214 |
|  |  | **six6** | 0.021 | 0.011 | 2 | 81.0 | 0.133 | 0.876 | 0.908 |
|  |  | **vgll3** | 0.500 | 0.250 | 2 | 81.0 | 3.150 | 0.048 | 0.169 |
|  |  | **Density:Sex** | 0.622 | 0.622 | 1 | 81.0 | 7.845 | 0.006 | 0.089 |
|  |  | **Density:six6** | 0.125 | 0.062 | 2 | 81.0 | 0.787 | 0.459 | 0.732 |
|  |  | **Density:vgll3** | 0.194 | 0.097 | 2 | 81.0 | 1.220 | 0.301 | 0.705 |
